# The Economic Burden of *Clostridioides difficile* in Denmark: A Retrospective Cohort Study

**DOI:** 10.3389/fpubh.2020.562957

**Published:** 2020-11-26

**Authors:** Uffe Christian Braae, Frederik Trier Møller, Rikke Ibsen, Steen Ethelberg, Jakob Kjellberg, Kåre Mølbak

**Affiliations:** ^1^Department of Infectious Disease Epidemiology and Prevention, Statens Serum Institut, Copenhagen, Denmark; ^2^i2minds, Aarhus, Denmark; ^3^Department of Public Health, Faculty of Health and Medical Sciences, University of Copenhagen, Copenhagen, Denmark; ^4^Danish National Institute for Local and Regional Government Research, Copenhagen, Denmark; ^5^Department of Veterinary and Animal Science, University of Copenhagen, Copenhagen, Denmark

**Keywords:** economic burden, hospital acquired infections, *Clostridium difficile*, cohort study, registries, CDI

## Abstract

**Objectives:** The aim of this study was to make a comprehensive economic assessment of the costs of hospital-acquired *C. difficile* infections (CDI).

**Methods:** We carried out a retrospective matched cohort study utilizing Danish registry data with national coverage to identify CDI cases and matched reference patients without CDI (controls) for economic burden assessment in Denmark covering 2011–2014. Health care costs and public transfer costs were obtained from national registries, and calculated for 1 year prior to, and 2 years after index admission using descriptive statistics and regression analysis.

**Results:** The study included 12,768 CDI patients and 23,272 matched controls. The total health care cost was significantly larger for CDI cases than controls throughout all periods. During the index admission period, cost was €12,867 per CDI case compared to €4,522 (*p* < 0.001) for controls, which increased to an average of €31,388 and €19,512 (*p* < 0.001) in Year 1 for the two groups, respectively. Excess costs were found both among infections with onset in hospitals and in the community. Diagnosis compatible with complications increased costs to on average >€91,000 per case. The regression analysis showed that CDI adds a substantial economic burden, but only explains about 1/3 of the crude difference observed in the matched analysis.

**Discussion:** The major economic impact of hospital-acquired CDI with complications underlines the importance of preventing complications in these patients. Our study provides an informed estimate of the potential economic gain per patient by successful intervention, which is likely to be relatively comparable across countries.

## Introduction

*Clostridioides difficile* is the leading cause of infectious diarrhea in hospitalized patients (1), and occurs regardless of economic development (2, 3). *C. difficile* causes toxin mediated colitis and often associated with a history of antibiotic treatment, increasing age and underlying illness, and exposure to health care systems (4, 5). *C. difficile* infection (CDI) has over the past decades been recognized as an increasing problem with rising mortality rates (6, 7). Due to the increase in frequency and severity of CDI, assessing the economic burden whereby policy and decision makers can make informed decisions on health care policy, is essential. CDI acquired at hospital or other health care facilities can be sub-classified as either healthcare-onset hospital-acquired (HOHA) or community-onset hospital-acquired (COHA). With the contemporary change in treatment paradigm toward shorter periods of hospitalization and more patients treated in ambulatory care, a change in the epidemiology of CDI with a shift from HOHA to COHA CDI would be expected (8). This hypothesis is corroborated by data from the national surveillance in Denmark (HAIBA annual report 2018, https://www.ssi.dk/-/media/arkiv/subsites/miba-og-haiba/dokument/haiba_2018-rsrapport.pdf?la=da), and this possible trend highlights the need for differentiation of the cost estimations for HOHA and COHA CDI. With the implementation of surveillance and more insight into the health burden of CDI, attention is drawn to improved prevention and control of CDI. To determine the cost-effectiveness of potential preventive measures and stimulate research, an economic assessment of the cost associated with CDI is needed. The aim of the present study was to assess the economic burden attributable to CDI during and after hospitalization for both HOHA and COHA CDI, using a population based matched cohort design.

## Methods

### Ethics Statement

The study was approved under the general agreement for non-interventional database studies between the Danish Data Protection Agency and Statens Serum Institut, reference number 2008-54-0474 and reported according to the STROBE statement (9).

### Study Design, Data Sources, and Participants

We carried out a retrospective matched cohort study utilizing Danish registry data with national coverage to acquire CDI cases and matched reference patients without CDI for economic burden assessment. The sampling population was the entire Danish population from 2011–2014, provided through the Civil Registry System (CRS) containing unique Personal Identification Numbers (PIN), which is linkable to various service records.

**Figure 1:**
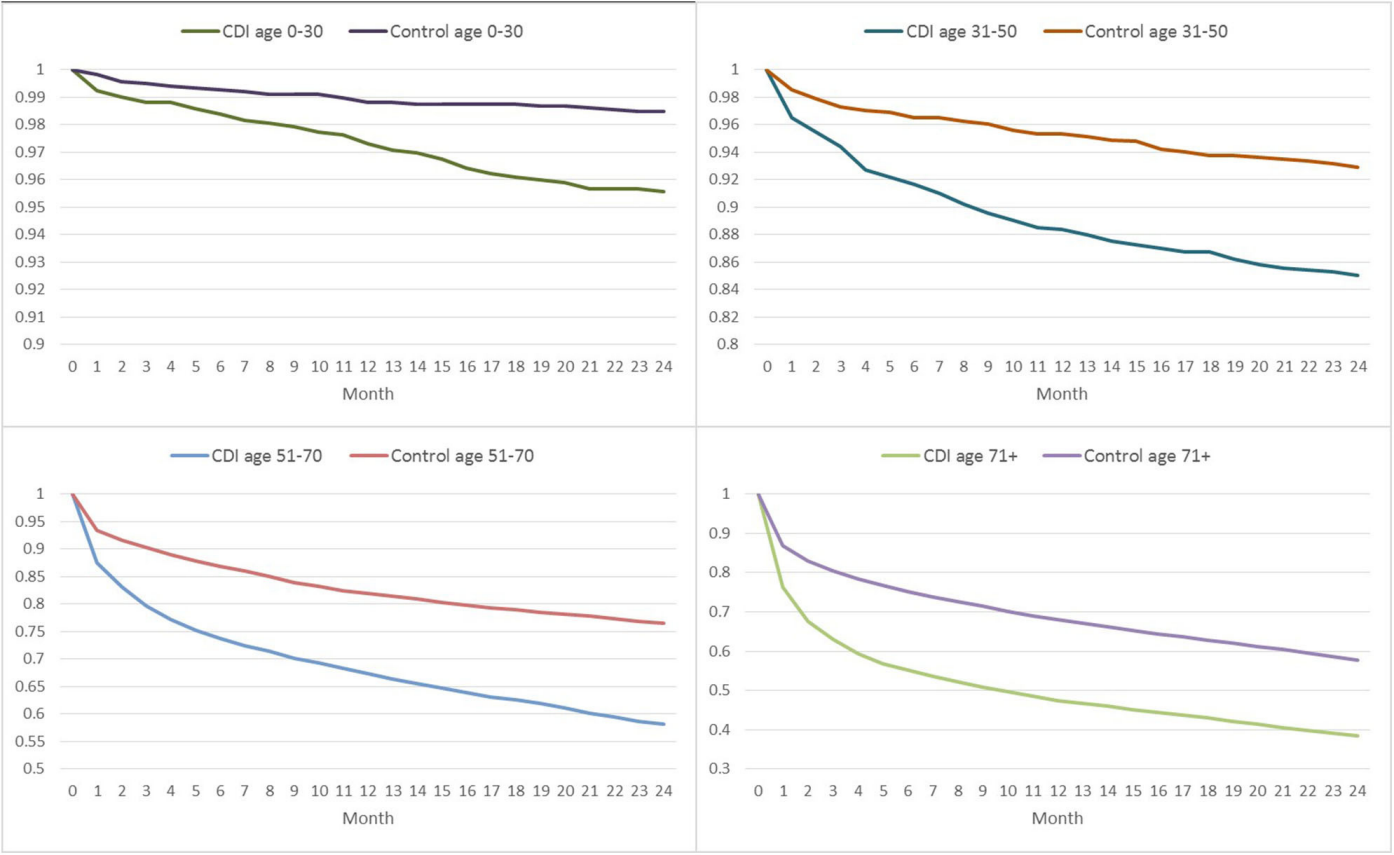
Flowchart illustrating case recruitment from the Hospital-Acquired Infections database (HAIBA) and the number of matched controls identified. CRS, Civil Registry System; PIN, Personal Identification Number; DNPR, Danish National Patient Registry; HOHA, healthcare-onset hospital-acquired; COHA, community-onset hospital-acquired; COCA, community-onset community-acquired.

We identified cases as patients with a confirmed first episode CDI, through the Hospital-Acquired Infections database (HAIBA). As part of the Danish surveillance system, CDI cases are registered in HAIBA and classified according to the ECDC case definition (10) with a few modifications (11). We distinguished cases as either healthcare-onset (HOHA) if a positive CDI-test was obtained ≥48 h after admission to a hospital and <48 h after discharge, or community-onset (COHA) if patients had a positive CDI-test between 48 h and 4 weeks after health care contact. Inclusion criteria to the case group were all CDI (HOHA and COHA) patients in HAIBA during 2011–2014 with a valid PIN (Figure 1). Patients with community-acquired CDI were excluded, as were patients where no matching control could be identified. All cases were linked with the Danish National Patient Registry (DNPR) (12) to obtain the action diagnosis along with a number of other variables (Supplementary Table 1).

The reference population (hereafter termed controls) were sampled from all admissions recorded during the study period in the DNPR and linked with the CRS to obtain sex and age, but excluding patients from HAIBA with CDI and those without a PIN. We matched each CDI case to two non-CDI controls, but cases with only one match were also retained. The controls were matched according to hospital, region, age, sex, action diagnosis, index year, and index month (that is the month of admission where the first CDI was registered). The action diagnosis was matched on the first three digits and age was matched in groups of intervals of 10 years until ≥81. Furthermore, an outpatient match was additionally performed for COHA patients with a pool of controls with no hospital admission 6 months prior to the end date of their ambulatory treatment. They were matched by sex, age, month, and year of their treatment end date. See the Supplementary Material for the full exclusion criteria and matching description.

### Periods for Cost Estimation

To determine the periods for cost estimations we used the index date in HAIBA as index, but for Year-1, Year 1, and Year 2 we used the dates from DNPR to match with the diagnosis-related groups in the Danish National Cost Database. This database provided the total cost for every in- and outpatient discharged from a public hospital in Denmark based on the patient's actual utilization of hospital services, with Diagnose Related Groups (DRG) for inpatients and Danish Ambulant Grouping System (DAGS) for outpatients. DRG-costs are linked to the discharge date and DAGS-costs are on the date of visit. Due to high mortality in the study population, the number of cases and controls declined over time. We therefore constructed the cost data over time as a gross dataset including all periods for all patients whether they were alive or not, but calculated the average cost of the patients by period, whilst taking the declining population into account. Apart from direct costs, we included derived costs to public transfer from the DREAM database containing all social transfer payments for all citizens in Denmark (13). Cost and employment data from after 2016 was unavailable at the time, so the analysis period, was restricted to 2010–2016, and for the study inclusion period to 2011–2014, to allow for 24 months follow up (Year 1, and Year 2) and 1 year and the pre-index period (Year -1).

### Cost Analysis

Cost data from DRG and DAGS were prices including rehabilitation. Index admission costs for HOHA were from the cost database. For transfer payment (any type of social welfare pay), we calculated the number of months, since we did not have prices for these transfers. Only transfers for people in the workforce were in the data, thus age and pension were unavailable. Complications were identified as the action diagnosis for outpatients and as the main action or secondary diagnosis during admission for inpatients. Patients only contributed cost until the date of death, as cost only occur while patients are alive. The cost were deflated to 2016 prices using the Danish Consumer Price index.

The first analysis included average cost and a test for significant difference between cases and controls. Since cases and controls were matched, we used a bootstrapped *t*-test with no additional explanatory variables in the model. As cost may be affected before onset of CDI, and CDI may result in long term complications, the costs where compared across different cost periods, to be able to compare cost over time. Cost were calculated for pre-index period (Year-1), index, and post-index period (Year 1 and Year 2). The pre-period was divided into two sub-periods, month−12 to −7 and month−6 to −1. The 24 months post-index period was split into the sub-periods month 1–3, month 4–6, month 7–9, month 10–12, and month 13–24. Patients were included if they were alive at least part of the sub-period, but excluded in future sub-periods after death occurred. Means of cost data for cases and controls was calculated with 95% confidence intervals (CI), with all costs adjusted to 2016 prices.

### Cost Regression

To control for residual confounding, we used a regression analysis for specific patients groups in order to determine the added cost of CDI. We used a 2-step gamma distributed analysis for all health cost (the sum of all types, inpatient somatic, outpatient somatic, inpatient psychiatric, outpatient psychiatric, primary sector, and prescription medication). A gamma distribution link rather than a logarithmic transformation was motivated by the occurrence of 0's in the cost variables, which did not follow a normal distribution (14). As independent variables we included the Charlson Co-morbidity Index in Year-1, i.e., the period prior to the diagnosis for cases and the corresponding index date for controls. In addition, we adjusted for sex, age, presence of inflammatory bowel disease (IBD), history of drug prescription (received more than four prescription drugs 12 months prior), and whether patients had been in hospital more than 7 days prior to index. This multivariate regression model estimated the total health costs attributed to CDI during the entire period and was run for CDI vs. controls, HOHA CDI vs. controls, and COHA CDI vs. controls. As a sensitivity analysis the model was re-run using data where cases corresponding to 10% of those with the highest and lowest cost before index and their controls were removed.

### Cost Analysis Stratified on Complications

Cost analysis stratified on complications were run to determine to what extent excess costs in patients could be related to the range of defined clinical complications. Complications were defined as having at least one of the following diagnoses in Year 1 or Year 2 (that is within 24 months after the index date, excluding the index admission): septicaemia, dehydration, renal failure, ileus, hypotension, shock, thromboembolic episodes, toxic megacolon, bowel perforation, colectomy, and isolation regimes. Cases with complications were allocated to the complication group for the whole study period and then stratified regardless of when the complication occurred. For a detailed description of the methodology of the matching and the cost analysis, please see the Supplementary Material.

## Results

### Matching Results and Population Descriptive

The study included 12,768 patients with hospital-acquired CDI and 23,272 matched controls (Table 1). Of the CDI cases, 7,183 were identified as HOHA and 5,585 cases as COHA, with 13,068 and 10,204 matched controls for each group, respectively. Most CDI patients were females (53.7%), and the mean age was 69.5 years with 33.6% of the cases being >80 years of age. The distribution of CDI cases across index years 2011–2014 were approximately equal (range: 3,384–3,036). Common diagnoses were diseases of the respiratory system (15.7%), factors influencing health status and contact with health services (14.0%), and certain infectious and parasitic diseases (12.0%).

**Table 1:** Descriptive statistics for *Clostridioides difficile* infection (CDI), healthcare-onset hospital-acquired (HOHA), and community-onset hospital-acquired (COHA) patients and matched controls at index time and prior to inclusion^a^.

	**All CDI patients**				**HOHA**				**COHA**			
	**CDI**		**Control**		**HOHA**		**Control**		**COHA**		**Control**	
**# Patients**, ***n***	12,768		23,272		7,183		13,068		5,585		10,204	
**Sex - Female**, ***n*****%**	6,860	53.7	12,530	53.8	3,783	52.7	6,901	52.8	3,077	55.1	5,629	55.2
**Age groups**, ***n*****%**												
0–10 years	478	3.7	859	3.7	166	2.3	285	2.2	312	5.6	574	5.6
11–20 years	177	1.4	310	1.3	54	0.8	88	0.7	123	2.2	222	2.2
21–30 years	270	2.1	496	2.1	64	0.9	112	0.9	206	3.7	384	3.8
31–40 years	307	2.4	547	2.4	111	1.5	188	1.4	196	3.5	359	3.5
41–50 years	460	3.6	801	3.4	222	3.1	376	2.9	238	4.3	425	4.2
51–60 years	1,049	8.2	1,918	8.2	576	8.0	1042	8.0	473	8.5	876	8.6
61–70 years	2,346	18.4	4,324	18.6	1,291	18.0	2,384	18.2	1,055	18.9	1,940	19.0
71–80 years	3,385	26.5	6,197	26.6	1,992	27.7	3,641	27.9	1,393	24.9	2,556	25.0
81+ years	4,296	33.6	7,820	33.6	2,707	37.7	4,952	37.9	1,589	28.5	2,868	28.1
**Age, mean (SD)**	69.5	21.0	69.3	20.7	72.7	18.0	72.6	17.6	65.5	23.7	65.1	23.4
**Index Year**, ***n*****%**												
2011	3,331	26.1	6,060	26.0	2,041	28.4	3,769	28.8	1,290	23.1	2,291	22.5
2012	3,066	24.0	5,600	24.1	1,801	25.1	3,295	25.2	1,265	22.6	2,305	22.6
2013	3,384	26.5	6,158	26.5	1,836	25.6	3,361	25.7	1,548	27.7	2,797	27.4
2014	2,987	23.4	5,454	23.4	1,505	21.0	2,643	20.2	1,482	26.5	2,811	27.5
**Region**, ***n*****%**												
Capital Region of Denmark	5,611	43.9	10,124	43.5	3,347	46.6	6,050	46.3	2,264	40.5	4,074	39.9
Region Zealand	2,463	19.3	4,594	19.7	1,529	21.3	2,828	21.6	934	16.7	1,766	17.3
Region of Southern Denmark	2,126	16.7	3,864	16.6	983	13.7	1,779	13.6	1,143	20.5	2,085	20.4
Central Denmark Region	1,501	11.8	2,795	12.0	731	10.2	1,344	10.3	770	13.8	1,451	14.2
North Denmark Region	1,067	8.4	1,895	8.1	593	8.3	1,067	8.2	474	8.5	828	8.1
**Medical history prior to inclusion**												
Hospitalization days 6 months prior, mean (SD)	16.3	21.3	6.1	13.3	17.8	21.9	7.8	14.7	14.3	20.2	3.9	11.0
Long-term hospitalization admissions 6 months prior (7+ days), *n*%	5,989	46.9	4,338	18.6	3,660	51.0	3,164	24.2	2,329	41.7	1,174	11.5
Charlson score 12 month prior, mean (SD)	1.4	1.8	0.9	1.6	1.5	1.8	1.1	1.7	1.3	1.8	0.7	1.4
**Match diagnosis**, ***n*****%**												
A Certain infectious and parasitic diseases	1,531	12.0	2,600	11.2	1,058	14.7	1,811	13.9	473	8.5	789	7.7
B A continued	37	0.3	56	0.2	19	0.3	26	0.2	18	0.3	30	0.3
C Neoplasms	1,132	8.9	2,027	8.7	513	7.1	927	7.1	619	11.1	1,100	10.8
D Neoplasms, blood diseases/blood-forming organs/certain immune disorders	213	1.7	359	1.5	88	1.2	145	1.1	125	2.2	214	2.1
E Endocrine, nutritional and metabolic diseases	455	3.6	837	3.6	267	3.7	495	3.8	188	3.4	342	3.4
F Mental and behavioral disorders	90	0.7	151	0.6	59	0.8	98	0.7	31	0.6	53	0.5
G Diseases of the nervous system	123	1.0	205	0.9	70	1.0	115	0.9	53	0.9	90	0.9
H Diseases of the eye and adnexa, Diseases of the ear and mastoid process	55	0.4	103	0.4	–		–		54	1.0	102	1.0
I Diseases of the circulatory system	1,073	8.4	2,019	8.7	747	10.4	1,409	10.8	326	5.8	610	6.0
J Diseases of the respiratory system	2,008	15.7	3,741	16.1	1,335	18.6	2,497	19.1	673	12.1	1,244	12.2
K Diseases of the digestive system	1,420	11.1	2,597	11.2	647	9.0	1,171	9.0	773	13.8	1,426	14.0
L Diseases of the skin and subcutaneous tissue	87	0.7	147	0.6	37	0.5	60	0.5	50	0.9	87	0.9
M Diseases of the musculoskeletal system and connective tissue	270	2.1	479	2.1	110	1.5	181	1.4	160	2.9	298	2.9
N Diseases of the genitourinary system	940	7.4	1,707	7.3	488	6.8	897	6.9	452	8.1	810	7.9
O Pregnancy, childbirth and the puerperium	28	0.2	54	0.2	8	0.1	16	0.1	20	0.4	38	0.4
P Certain conditions originating in the perinatal period	9	0.1	17	0.1	0		0		5	0.1	9	0.1
Q Congenital malformations, deformations and chromosomal abnormalities	40	0.3	75	0.3	23	0.3	43	0.3	17	0.3	32	0.3
R Symptoms, signs/abnormal clinical and laboratory findings, not elsewhere classified	619	4.8	1,160	5.0	293	4.1	548	4.2	326	5.8	612	6.0
S Injury, poisoning and certain other consequences of external causes	492	3.9	903	3.9	292	4.1	529	4.0	200	3.6	374	3.7
T S continued	359	2.8	654	2.8	228	3.2	416	3.2	131	2.3	238	2.3
Z Factors influencing health status and contact with health services	1,787	14.0	3,381	14.5	896	12.5	1,675	12.8	891	16.0	1,706	16.7
**IBD 12 month prior**, ***n*****%**												
K50_12M_prior	160	1.3	150	0.6	53	0.7	65	0.5	107	1.9	85	0.8
K51_12M_prior	305	2.4	166	0.7	117	1.6	75	0.6	188	3.4	91	0.9
K52_12M_prior	365	2.9	240	1.0	186	2.6	143	1.1	179	3.2	97	1.0
A0_12M_prior	831	6.5	435	1.9	417	5.8	307	2.3	414	7.4	128	1.3
**Share any of the above IBD**^**b**^	1,468	11.5	919	3.9	688	9.6	555	4.2	780	14.0	364	3.6
**Number of prescription drug ATC-codes 7 digits 12 month prior**, ***n*****%**												
0	313	2.5	991	4.3	195	2.7	442	3.4	118	2.1	549	5.4
1–5	2,240	17.5	6,019	25.9	1223	17.0	2848	21.8	1017	18.2	3,171	31.1
6–9	2,684	21.0	5,547	23.8	1505	21.0	3078	23.6	1179	21.1	2,469	24.2
10+	7,531	59.0	10,715	46.0	4260	59.3	6700	51.3	3271	58.6	4,015	39.3
**Number of prescription drug ATC-codes 4 digits 12 month prior**, ***n*****%**												
0	313	2.5	991	4.3	195	2.7	442	3.4	118	2.1	549	5.4
1–5	2,605	20.4	6,744	29.0	1405	19.6	3240	24.8	1200	21.5	3,504	34.3
6–9	3,190	25.0	6,325	27.2	1818	25.3	3565	27.3	1372	24.6	2,760	27.0
10+	6,660	52.2	9,212	39.6	3765	52.4	5821	44.5	2895	51.8	3,391	33.2
**Number of prescription drug ATC-codes 3 digits 12 month prior**, ***n*****%**												
0	313	2.5	991	4.3	195	2.7	442	3.4	118	2.1	549	5.4
1–5	3,066	24.0	7,670	33.0	1661	23.1	3737	28.6	1405	25.2	3,933	38.5
6–9	3,880	30.4	7,253	31.2	2236	31.1	4229	32.4	1644	29.4	3,024	29.6
10+	5,509	43.1	7,358	31.6	3091	43.0	4660	35.7	2418	43.3	2,698	26.4

a Distributions do not always amount to 100%, as cells with <5 observations are not listed due to anonymity requirements.

b *ICD-10 = K50, K51, K52, A0*.

Prior to the index date, cases and matched controls were incomparable on a number of parameters. Cases had on average spent 16.3 days in hospital compared with 6.1 days spent among the controls. The share of long-term hospitalization admissions (7+ days) 6 months prior was 46.9 and 18.6% among the cases and the controls, respectively. The Charlson score 12 months prior to inclusion was on average 1.4 and 0.9 for cases and controls, respectively. Among the cases, 11.5% had a diagnosis of IBD 12 months prior to inclusion, whereas 3.9% of the controls had IBD. Taken together, even with a careful matching procedure based on underlying disease codes, cases of CDI had an excess of severe illness and were hospitalized longer, and had higher frequencies of IBD before index date. Thus, matching did not account for the differences in morbidity between the cases and controls.

### Complications and Mortality

Typical complications for COHA and HOHA were septicaemia, renal failure, dehydration, and isolation regimes (Supplementary Table 2). During Year 1, 8.1% cases had more than one complication, with septicaemia and renal failure causing most days in hospital among both COHA and HOHA cases. The complications among CDI patients accounted for a large share of the days spent in hospital. COHA CDI patients were on average hospitalized 17.7 days during Year 1, with 34.6% attributed to complications, and 8.0 days during Year 2, with 32.2% attributed to complications. HOHA CDI patients were in comparison hospitalized on average 21.7 days during Year 1, with 32.6% attributed to complications, and 10.5 days during Year 2, with 32.4% due to complications (Supplementary Table 3).

**Figure 2:**
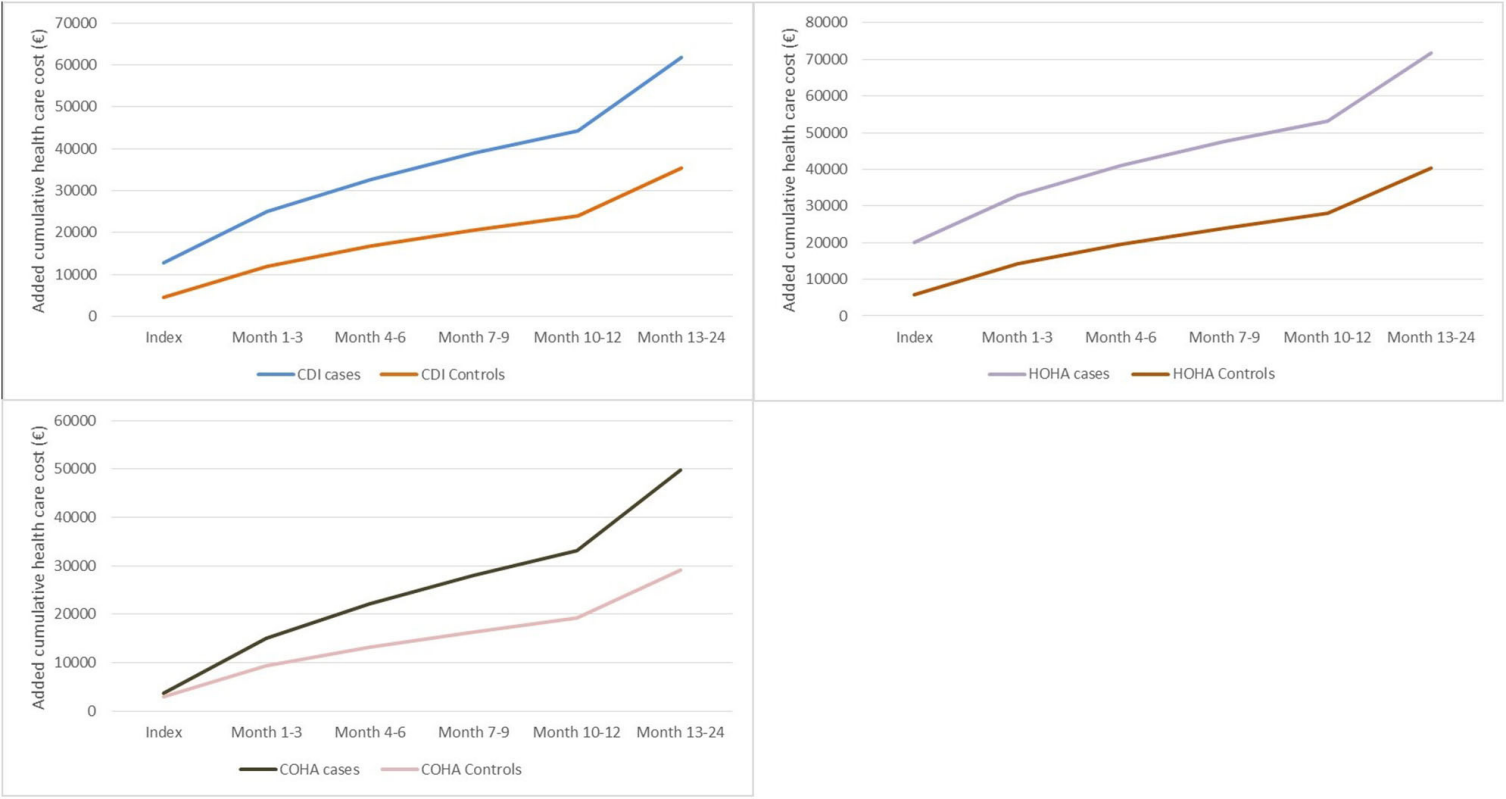
Survival months of patients with *Clostridioides difficile* infection (CDI) and controls in different age groups, based on Kaplan Meier analysis for each age group.

The mortality among CDI patients was high, especially among the age group 71+ years, where 38% survived until 24 months, compared to 58% of the matched controls (Figure 2). The difference in survival between CDI patients and the controls became less apparent with declining age. In general, COHA cases had better survival than HOHA cases, e.g., a 21.7% survival among 71+ HOHA compared with 31.0% among COHA after 72 months (Supplementary Figures 1, 2). A similar magnitude of difference in survival was seen among the matched controls which may indicate that the relative mortality was independent of onset of CDI.

### Cost of *C. difficile* Infections

**Figure 3:**
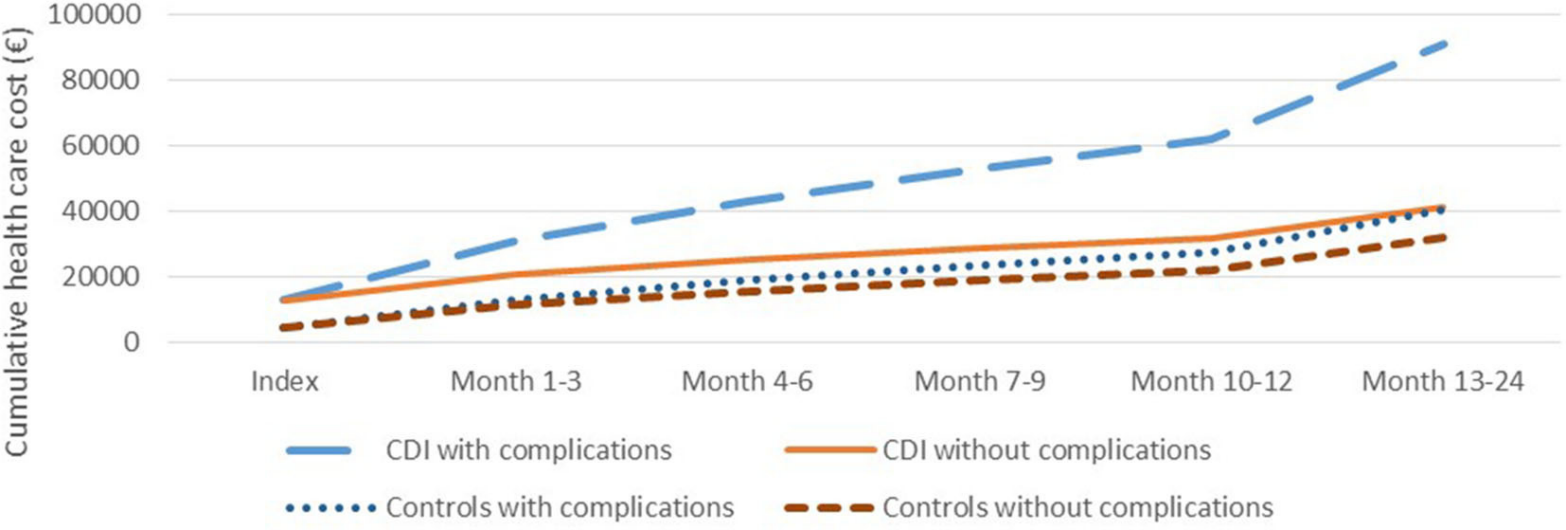
The mean cumulative health care cost over time of cases with *Clostridioides difficile* infection (CDI), COHA CDI, and HOHA CDI, and matched controls for each of the three groups.

The total health care cost was significantly larger for CDI cases compared to the controls throughout all periods studied, including Year-1 before diagnosis (Table 2). A full breakdown of all costs for CDI, COHA, and HOHA, is available in the Supplementary Tables 5.1–5.9. During month−12 to −7 the average cost of CDI cases was €8,700 compared to €6,073 (*p* < 0.001) for the controls, which increased to €21,795 compared to €10,188 (*p* < 0.001) for the two groups during month−6 to −1. At index, cost was €12,867 for cases compared to €4,522 (*p* < 0.001) for controls. In Year 1, the cost was €31,388 for CDI and €19,512 for the controls (*p* < 0.001), and €17,590 and €11,260 (*p* < 0.001) for the two groups, respectively in Year 2. During the index period and Year 1 and 2, CDI cases spent on average 1.1 month longer on public transfer income compared to the controls (*p* < 0.001). We found increased costs both for COHA and for HOHA CDI cases compared with the matched controls (Table 2). At index the mean cost of a COHA CDI case was €3,704 compared to €2,996 (*p* < 0.001) for a control, and €16,640 vs. €9,850 (*p* < 0.001) during Year 2 for cases and controls, respectively. HOHA CDI cases on average were estimated to cost €19,992 at index compared to €5,714 (*p* < 0.001) for controls. During Year 2 the costs were estimated to €18,553 and €12,523 (*p* < 0.001) for cases and controls, respectively. Based on the cumulative health care cost from index to Year 2, HOHA CDI cases were more costly with a mean economic burden of >€70,000, whereas, a COHA CDI case had a mean economic burden of ~€50,000 (Figure 3).

**Table 2:** CDI health care costs of all CDI patients, COHA, HOHA, and their matched controls.

	**Health cost CDI**					**Health cost COHA**					**Health cost HOHA**				
	**# Patients**		**Total health cost**			**# Patients**		**Total health cost**			**# Patients**		**Total health cost**		
	**CDI**	**Control**	**CDI**	**Control**	*P*-value^*^	**COHA**	**Control**	**COHA**	**Control**	*P*-value^*^	**HOHA**	**Control**	**HOHA**	**Control**	*P*-value^*^
**Period**	***n***	***n***	**€**	**€**		***n***	***n***	**€**	**€**		***n***	***n***	**€**	**€**	
Year−1 (-1 to−12 month)	12,768	23,272	30,494	16,261	<0.001	5,585	10,204	29,613	12,873	<0.001	7,183	13,068	31,180	18,906	<0.001
Month−12 to−7)	12,786	23,272	8,700	6,073	<0.001	5,585	10,204	9,114	5,327	<0.001	7,183	13,068	8,377	6,655	<0.001
Month−6 to−1)	12,786	23,272	21,795	10,188	<0.001	5,585	10,204	20,499	7,546	<0.001	7,183	13,068	22,802	12,251	<0.001
Index cost^**^	12,768	23,272	12,867	4,522	<0.001	5,585	10,204	3,704	2,996	<0.001	7,183	13,068	19,992	5,714	<0.001
Month 1–3	11,846	22,134	12,102	7,498	<0.001	5,571	9,868	11,244	6,267	<0.001	6,275	12,266	12,864	8,487	<0.001
Month 4–6	9,196	19,873	7,742	4,716	<0.001	4,502	9,092	7,187	3,887	<0.001	4,694	10,781	8,274	5,416	<0.001
Month 7–9	8,366	18,901	6,200	3,826	<0.001	4,140	8,768	5,861	3,203	<0.001	4,226	10,133	6,533	4,366	<0.001
Month 10–12	7,883	18,184	5,344	3,472	<0.001	3,938	8,515	5,081	2,928	<0.001	3,945	9,669	5,607	3,951	<0.001
Year 2 (month 13–24)	7,512	17,580	17,590	11,260	<0.001	3,782	8,305	16,640	9,850	<0.001	3,730	9,275	18,553	12,523	<0.001

* P-value from t-test and bootstrapping.

** *Index admissions are calculated from the cost database for HOHA. Since COHA is partly outpatient onset, their index admission is from DRG*.

### Economic Burden of CDI

**Figure 4:**
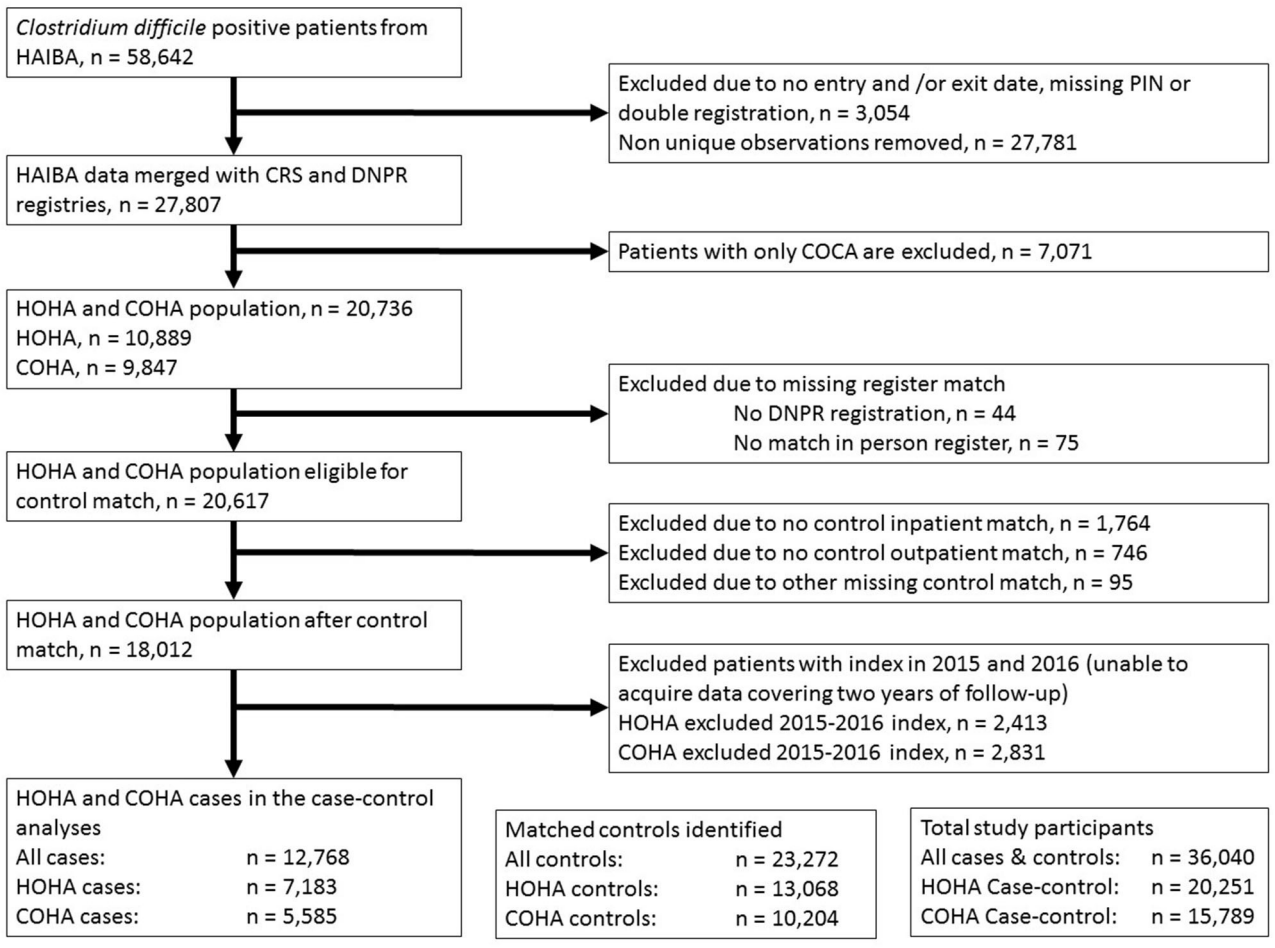
The mean cumulative health care cost over time of cases with *Clostridioides difficile* infection (CDI) with and without complications and the matched controls.

The largest share of the economic burden was related to complications in combination with CDI, which after 2 years on average resulted in an economic burden of more than €91,000 per case compared to ~€40,000 among the matched controls. In comparison, cases without complications incurred an economic burden after 2 years of ~€41,000 whereas the matched controls incurred an economic burden of ~€32,000 (Figure 4).

The regression model (see Tables 3, 4 for the parameters) showed that over a 2-year period, 33% of the economic burden among patients with CDI could be ascribed to CDI. Among patients with HOHA CDI, 37% of the economic burden could be explained by CDI, whereas 23% of the economic burden could be attributed to CDI among COHA CDI patients. Table 3 shows the estimated health care costs of typical patients groups during different periods and the estimated potential gained monetary value by preventing CDI. The largest attribution to cost by CDI was observed during index and the 1st year of disease. From the estimated cost in specific patients groups, the model predicted that in patients with severe disease, a smaller proportion of the cost could be attributed to CDI in comparison to “healthier” patients (Table 5). The sensitivity analysis showed that there was no significant impact by removing the 10% of the data covering outliers in either end (data not shown).

**Table 3:** Regression total health cost including all patients alive at least part of year 1 (including index date).

**Population**	***N***	**Year 1**	**Total health cost Year 1**				
			**Estimate**	**Std. Error**	**LCL 95%**	**UCL 95%**	***P*-value**
**CDI, all included**		**CDI, all included**					
CDI (*N*)	12,768	CDI vs. control	0.47	0.01	0.44	0.49	<0.001
Control (*N*)	23,272	**Controlled for**					
		Charlson Comorbidity Index (CCI)	0.14	0.00	0.14	0.15	<0.001
		Gender (male)	0.18	0.01	0.16	0.20	<0.001
		Age	−0.07	0.00	−0.08	−0.07	<0.001
**HOHA, all included**		**HOHA, all included**					
HOHA (*N*)	7,183	HOHA vs. control	0.50	0.01	0.47	0.53	<0.001
Control (*N*)	13,068	**Controlled for**					
		Charlson Comorbidity Index (CCI)	0.09	0.00	0.08	0.10	<0.001
		Gender (male)	0.15	0.01	0.12	0.18	<0.001
		Age	−0.16	0.00	−0.17	−0.15	<0.001
**COHA, all included**		**COHA, all included**					
COHA (*N*)	5,585	COHA vs. control	0.36	0.02	0.33	0.40	<0.001
Control (*N*)	10,204	**Controlled for**					
		Charlson Comorbidity Index (CCI)	0.21	0.01	0.19	0.22	<0.001
		Gender (male)	0.17	0.02	0.14	0.20	<0.001
		Age	−0.02	0.00	−0.03	−0.01	<0.001

**Table 4:** Regression total health cost including all patients alive all of Year 1 and at least part of Year 2.

**Population**	***N***	**Year 1**	**Total health cost Year 1**				
			**Estimate**	**Std. Error**	**LCL 95%**	**UCL 95%**	***P*-value**
**CDI alive Year 1**		**CDI alive Year 1**					
CDI (*N*)	7,512	CDI vs. control	0.56	0.01	0.53	0.59	<0.001
Control (*N*)	17,580	**Controlled for**					
		Charlson Comorbidity Index (CCI)	0.26	0.01	0.25	0.27	<0.001
		Gender (male)	0.19	0.01	0.16	0.21	<0.001
		Age	−0.04	0.00	–0.05	–0.04	<0.001
**HOHA alive Year 1**		**HOHA alive Year 1**					
HOHA (*N*)	3,730	HOHA vs. control	0.63	0.02	0.59	0.67	<0.001
Control (*N*)	9,275	**Controlled for**					
		Charlson Comorbidity Index (CCI)	0.19	0.01	0.17	0.20	<0.001
		Gender (male)	0.16	0.02	0.12	0.19	<0.001
		Age	−0.12	0.00	–0.13	–0.11	<0.001
**COHA alive Year 1**		**COHA alive Year 1**					
COHA (*N*)	3,782	COHA vs. control	0.44	0.02	0.40	0.48	<0.001
Control (*N*)	8,305	**Controlled for**					
		Charlson Comorbidity Index (CCI)	0.32	0.01	0.31	0.34	<0.001
		Gender (male)	0.18	0.02	0.14	0.22	<0.001
		Age	–0.01	0.00	–0.01	0.00	0.067
		**Year 2**					
		**CDI alive Year 1**					
		CDI vs. control	0.28	0.01	0.25	0.31	<0.001
		**Controlled for**					
		Charlson Comorbidity Index (CCI)	0.29	0.01	0.28	0.30	<0.001
		Gender (male)	0.18	0.01	0.16	0.21	<0.001
		Age	−0.02	0.00	–0.03	–0.02	<0.001
		**HOHA alive Year 1**					
		HOHA vs. control	0.23	0.02	0.19	0.27	<0.001
		**Controlled for**					
		Charlson Comorbidity Index (CCI)	0.24	0.01	0.23	0.26	<0.001
		Gender (male)	0.13	0.02	0.10	0.17	<0.001
		Age	−0.09	0.01	–0.10	–0.09	<0.001
		**COHA alive Year 1**					
		COHA vs. control	0.34	0.02	0.30	0.38	<0.001
		**Controlled for**					
		Charlson Comorbidity Index (CCI)	0.34	0.01	0.32	0.36	<0.001
		Gender (male)	0.21	0.02	0.17	0.24	<0.001
		Age	0.02	0.00	0.01	0.03	<0.001

**Table 5:** Total cost and the added cost of CDI among selected patient groups from CDI, CDI HOHA, and CDI COHA.

	**Per case economic burden**, €					**Monetary gain per case of CDI prevention**, €**(%)**				
	**Index and part of Year 1**		**Year 1**		**Year 2**	**Index and part of Year 1**		**Year 1**		**Year 2**
***CDI patients***										
Women aged 51–60	24,948		22,343		6,983	8,214 (33)		8,362 (37)		1,105 (16)
Women aged 51–60 with a co-morbidity score 2	31,373		33,773		11,406	10,329 (33)		12,640 (37)		1,805 (16)
Woman aged 51–60 with a co-morbidity score 2 and 7+days in hospital prior	48,810		58,422		17,018	20,055 (41)		24,329 (42)		4,654 (27)
Woman aged 51–60 with a co-morbidity score 2, ATC5, and 7+days in hospital prior	42,291		51,886		23,477	6,681 (16)		8,384 (16)		2,258 (10)
***HOHA CDI patients***										
Women aged 51–60	38,892		35,419		9,113	14,372 (37)		15,373 (43)		1,474 (16)
Women aged 51–60 with a co-morbidity score 2	45,384		48,556		13,879	16,772 (37)		21,075 (43)		2,245 (16)
Women aged 51–60 with 7+days in hospital prior and ATC5	44,274		44,378		15,604	10,261 (23)		11,281 (25)		1,294 (8)
Women aged 51–60 with 7+days in hospital prior, ATC5, and IBD	36,419		35,405		16,882	3,509 (10)		2,650 (7)		−2,216 (-13)
***COHA CDI patients***										
Women aged 41–50	15,827		14,265		5,798	3,576 (23)		3,732 (26)		845 (15)
Women aged 41–50 with a co-morbidity score 1.5	20,174		25,293		8,804	4,558 (23)		6,617 (26)		1,283 (15)
Women aged 41–50 with a co-morbidity score 1.5 and ATC5	25,358		27,201		15,145	5,825 (23)		7,213 (27)		2,035 (13)
Women aged 41–50 with a co-morbidity score 1.5 and IBD	23,157		24,373		11,197	7,858 (34)		8,162 (33)		2,151 (19)

*Estimations are based on regression and with interactions if the terms +7 days in hospital, ATC5 (received five prescription drugs), or IBD occurs and includes both direct and indirect costs. The period “part of Year 1” includes patients that died during this period. A proportionately identical difference was observed for men (data not shown)*.

## Discussion

Although cases and controls were carefully matched on several variables including age and diagnosis, we found considerable differences in clinical parameters and hospital costs between cases and controls even before the diagnosis of CDI. This underscores that CDI is a challenge of the frailest patients, and that it is critical to address confounding in epidemiological and economic studies of CDI. It is, however, also possible that some of the difference in Year−1 can be ascribed to a delayed diagnosis of CDI. Interestingly the cost difference was less during the months−12 to −7 than during the months−6 to −1 leading to the index; potentially indicating that CDI had manifestations well before diagnosis in some patients. To address confounding, we conducted a regression analysis, which confirmed that CDI adds a substantial economic burden, but only explains about 1/3 of the crude difference observed in the matched analysis. The conducted sensitivity analysis showed that the model accounted well for the cost differences between cases and controls prior to index. The cost difference between cases and controls were more than four times higher in CDI cases with complications than cases without complications. The association between clinical complications and excess costs adds additional weight to the plausibility of the results.

We found excess costs both in HOHA and COHA CDI patients, but we also found that the costs of COHA was less than among HOHA. While this difference was expected, we believe that our study is the first to address this question. The differences in the economic health care burden of HOHA and COHA underlines the importance of acknowledging the distinction of the two groups in economic health care burden analyses, in particular because COHA will be relatively more important as health care reform progresses toward more ambulatory care.

There is limited consensus about the definition of CDI, populations studied, the designs, and range of cost included. This complicates an overall comparison between our study and previous published literature. A recent review of the economic burden of CDI from different countries found that the attributable mean CDI costs ranged from $8,911 to $30,049 for hospitalized patients (2014 USD) (15). Although direct comparisons are difficult, our estimates for the health care costs of CDI patients appear well outside this range as the average CDI case in Denmark, with the inclusion of the 2 year post-period, cost ~€61,800 in 2016, equivalent to $68,000 USD. However, higher estimates are often seen in health economics when using high quality registry data with broad coverage due to the potential capability of capturing more of the actual costs (16).

A recent retrospective analysis using individual-level data from from Apr. 1, 2005 to Mar. 31, 2015, in Ontario Canada health databases, found the median costs attributable to C. difficile infection to be $1051 for that associated with long-term care facilities, $13 249 for community-associated infection and $11 917 for ACH-associated/community-onset infection (17).

A study from Germany estimating both direct and indirect cost of CDI in hospitalized patients found CDI to cost €18,460 (18), which is comparable to the index cost of €19,992 for HOHA patients found in this study. A study from Australia that investigated CDI in a population aged 45 years and up (19), found that patients with cardiovascular disease cost on average $17,947 ± 2,611 with CDI and $7,825 ± 46.7 without CDI (difference of AUS $10,122 ~ €6,230). This is comparable to the difference in cost at index (€8,345) between a CDI case (€12,867) and a control (€4,522) in our study.

Nanwa et al. (20) conducted an incidence-based propensity-score matched cohort study to evaluate costs attributable to hospital-acquired CDI from the healthcare payer perspective. They found that hospital-acquired CDI was associated with worse clinical outcomes in patients compared to clinical outcomes in matched uninfected patients. Similarly, our study reported higher mortality among cases compared to the matched controls (Figure 2). Nanwa et al. (20) also reported that the attributable costs were greatest during the index hospitalization, but decreased over time albeit higher costs persisted compared to matched non-CDI patients. This is comparable to the results presented here for hospitalized patients, but for COHA CDI patients the largest economic burden was incurred in Month 1–3 after treatment.

McGlone et al. (21) developed an economic computational model to determine the annual cost of healthcare-acquired CDI in the Unites States. The model incorporated hospital-acquired CDI associated costs in regards to hospital, third-party payer, and societal perspectives. Most costs incurred were during a patient's primary CDI episode, with an estimated cost of as much as $12,607 (2011 USD). With the regression models developed during the present study, we can estimate the economic burden of various patient groups with hospital-acquired CDI, and the potential to develop the model further to quantify the total economic burden of all hospital-acquired CDI patients in Denmark exist.

Our study is subject to limitations. Although clinical outcomes are representative of the clinical settings in other high-income countries, data on direct costs and public transfer are to a large degree specific to the Danish society. Nonetheless, the results do have general international applicability due to the uniqueness of the data quality, the large range of outcomes, the large sample size, and the potential to be modified to other settings and used in modeling burden of CDI in other countries. We had no detailed clinical data on comorbidity, which represents another limitation. However, the use of a comorbidity score and adjustment for history of medications is thought to have adjusted for confounding. This is supported by the observation that IBD (which results in increased sampling of fecal specimens and therefore an increased chance of diagnosis of CDI) were not associated with increased costs in the regression analysis. Finally, complications were defined based on data from administrative registries and not a detailed clinical assessment, which was beyond the scope of this study.

In conclusion, our study estimates the attributable economic burden of hospital-acquired CDI in Danish patients and provides an informed estimate of the potential economic gain per patient by successful intervention. We emphasize the need to include COHA CDI in order to make comprehensive estimates of the overall economic burden of CDI on health care systems. At present, only few national surveillance systems have the capacity to disentangle HOHA from COHA, and often COHA cases may be ignored because the onset is in the community where they may not be categorized as hospital-acquired cases. Furthermore, the results highlight the importance of identifying and preventing complications in patients with hospital-acquired CDI, and the need to investigate strategies to prevent CDI in susceptible patients.

## Data Availability Statement

The datasets presented in this article are not readily available because the authors confirm that, for approved reasons, some access restrictions apply to the data underlying the findings. We used population-register based data including personal identifiers. To access this data it is necessary to apply to the Danish National Board of Health by completing an extensive data approval application.

## Author Contributions

RI and JK generated and analyzed the data. UB, FM, SE, and KM interpreted the data. UB wrote the first manuscript draft. All authors critically reviewed the manuscript.

## Conflict of Interest

RI was employed by the company i2minds. The remaining authors declare that the research was conducted in the absence of any commercial or financial relationships that could be construed as a potential conflict of interest.
