# Supplementary material for: The Economic Burden of Clostridioides difficile in Denmark: A Retrospective Cohort Study
Source: Front Public Health. 2020 Nov 26;8:562957. doi: 10.3389/fpubh.2020.562957 (PMC7725905; doi:10.3389/fpubh.2020.562957)
Supplement: Supplementary file 1 [file Data_Sheet_1.docx]

**Supplementary Material**

**The economic burden of *Clostridioides difficile* in Denmark: a retrospective cohort study**

Contents

[Data sources 3](#_Toc27386816)

[Supplementary Table 1 3](#_Toc27386817)

[Cost estimation 4](#_Toc27386818)

[Periods for costs 4](#_Toc27386819)

[The Cost Database 4](#_Toc27386820)

[Analysis period 5](#_Toc27386821)

[Complications after CDI 5](#_Toc27386822)

[Supplementary Table 2 7](#_Toc27386823)

[Supplementary Table 3 8](#_Toc27386824)

[Supplementary Table 4.1 9](#_Toc27386825)

[Supplementary Table 4.2 10](#_Toc27386826)

[Cost analysis 11](#_Toc27386827)

[Supplementary Table 5.1 12](#_Toc27386828)

[Supplementary Table 5.2 12](#_Toc27386829)

[Supplementary Table 5.3 12](#_Toc27386830)

[Supplementary Table 5.4 13](#_Toc27386831)

[Supplementary Table 5.5 13](#_Toc27386832)

[Supplementary Table 5.6 13](#_Toc27386833)

[Supplementary Table 5.7 13](#_Toc27386834)

[Supplementary Table 5.8 14](#_Toc27386835)

[Supplementary Table 5.9 14](#_Toc27386836)

[Cost Regression 15](#_Toc27386837)

[Cost analysis stratified on complications 15](#_Toc27386838)

[Supplementary Table 7.1 16](#_Toc27386839)

[Supplementary Table 7.2 16](#_Toc27386840)

[Supplementary Table 7.3 17](#_Toc27386841)

[Survival analysis 18](#_Toc27386842)

[Supplementary Table 8 18](#_Toc27386843)

[Supplementary Figure 1 19](#_Toc27386844)

[Supplementary Figure 2 20](#_Toc27386845)

# Data sources

Supplementary Table 1. Data sources with obtained variables, all of which are linkable using the Danish unique personal identification number (PIN)

| **Registry data sources** | **Purpose of access** | **Variables** |
| --- | --- | --- |
| Civil Registry System (CRS) | Containing unique personal identification numbers (PIN) enabling data linking with other registries | Date of birth, Death date, Sex |
| Hospital Acquired Infections database (HAIBA) | The identification of HOHA and COHA CDI patients | COHA, HOHA, Index month and year |
| Danish National Patient Registry (DNPR) | For the assessment of diagnosis and procedures, including cause of admission, complications, and comorbidity for both in- and outpatients | Action diagnosis, Hospital, Admission date, Discharge date, Patient type, Days of admission, Outpatient treatment date, Complications, Comorbidity |
| Danish National Cost Database | Provides the total cost for every patient discharged from a public hospital in Denmark based on the patient’s actual utilization of hospital services (DRG (inpatient) and DAGS (outpatient)). | Total inpatient price, Total outpatient price |
| Danish registry for time spend on public transfer income (DREAM) | Time spend on public transfer income | Type of social income, Start date, End date |

# Cost estimation

### Periods for costs

To calculate costs for each individual,index dates were extracted from HAIBA along with pre- and post-periods from DNPR, to enable match with DRG and DAGS. DRG-costs (inpatient) were on outdate and DAGS were on the date of visit (outpatient).

Due to high mortality, the share of patients that diedduring the post periods were high, thus populationsof both cases and controls declined over time. Hence,data were constructed over time as a gross dataset including all periods regardless of status(alive/dead/emigrated). From this dataset we were able toexclude all observations, where a patient died in the previous period. Thesecond dataset included patients that were alive for part or the entirety of a specific period.

HOHA: The index date was the exit-date of the admission (for cases and controls individually). All admissions (DRG) with a valid exit-date in the 12 months pre-period (before the in-date=index date) were included in the pre-period costs.All admissions (DRG) with a valid exit-date in the post periods (3, 6, 12, 24 months) were included in the post costs.

Outpatient visits (DAGS) before the index-date were defined aspre-period, and visits after index date was defined as post-period.

### The Cost Database

The Cost Database was only used to calculate the cost ofhospital admissions, and referred to index admissions for HOHA-patients and their controls. The Cost Database did not match the HOHA patient data completely, but 95% had a direct match on the admission date. There were 316 patientswith more than one observation in the Cost Database with the same in-date, outdate, and hospital. For some of these, one of the observations was for emergency room (ER)visits. Such observations weregrouped into one observation with all costs for the duration of the admission. We summed the admission costsboth including and excludingER costs. In the final analysis, we excluded cost related to ER visits.

To include the 5% of HOHA patients, that were not found in the Cost Database, we ran a multiple imputation (MI) on log cost, and a simple regression to estimate the significance of the difference concerning cost for cases and controls. Missing observations and observations with cost=0 were imputed. The imputation was run 25 times, and in the cost analysis,the average of the imputed cost were used for the patients with no information or cost=0.The variable used from the Cost Database wasv_tot_cost.

Some admission registrations had an index date (from DNPR) not matchingthe exit-date with total cost in DRG-data. This was because we matched at the index time for the cost of illness (COI), of a specific period in DNPR, and thus,not necessarily the whole period when total cost was allocated in DRG. When replacing DRG-cost with cost from the Cost Database, 86% matched on index-date. Correspondingly we replaced the DRG-cost with cost from the Cost Database for these observations. For the remaining 14% we matched in several steps. First we matched with the closest DRG-cost after the index date, and subtracted the v_pris (daily rate for the index admission) from the later DRG-cost (from collapsed admissions).

New_drg_cost=drg_cost-v_pris_index

For some admissions the DRG-cost was availableearlier than the index-admission, and we then subtracted the v_pris_index from the earlier DRG-cost.The reason for only subtracting v_priswas that the index admission could have been part of a longer admission, and the cost from the cost database,in that case, would only cover part of the entire admission. No subtractionswere made for 23 unmatched observations.

COHA admissions: The index date for cases and controls were the exit-date for the admission used to match case and control. The admission closest to the index date (before index date from HAIBA) for cases was used.This ensured that the admission linked to COHA always was in the post-period (or index-period) for both case and controls.

COHA outpatient: One group of COHA outpatients had an index date within 90 days of the exit-date of an outpatient record (outpatient records in DNPR runs from start to end with dates for start and end over all visits rather than for the single visits). For this group we used the same method as for the HOHA/COHA admissions above, as we used the exit-date for the outpatient record as index date. An outlier with an index date of 127 days after the enddate of and outpatient record was deleted.The other group had an index date (from HAIBA) between the in-date and out-date for outpatients and the HAIBA index date were matched to the exit-date for the outpatients. Providingthe controls with an out-date as index date would mean a relative higher cost burden in the pre-period for controls by construction, since all cost for the outpatient visits until the out-date would be in the pre-period, while the cases wouldhave some of the visits (and therefore costs) in the post-period. For this group the d_uddtowas used as the index date for controls even though there could be a problem for outpatient costs being lopsided to the pre-period for controls. To control for this the group was split in the analysis.

### Analysis period

The study inclusion period was2011-2014. The analysis included a pre-period of 12 months and a post-period of 24 months. Since cost and employment data were available for 2010 we included the year 2011 in the analysis. However, cost and employment data after 2016 were unavailable, so the timeframe of the analysis including a 24 months post-period wasrestricted to 2010-2016.

The pre-period excluded index date. The post period included the index date except when we only included HOHA. For the HOHA analysis the index admission period was separated.

### Complications after CDI

The analysis of complications after CDI included

- Number of complications (Supplementary Table 2)
- Hospitalization due to complications (Supplementary Table 3)
- Cost (inpatient-outpatient) due to complications (Supplementary Table 4.1 & 4.2)

In Year 1 and Year 2 after index date only patients thatwere alive at least part of the period were included. Thus, patients that died during the index admission were excluded, and patients that died during Year 1 were excluded fromYear 2.

Complications were identified for inpatients as the main diagnosis, action diagnosis,or secondary diagnosis during an admission. Cost and hospitalised days were found at the end of the admission, when the DRG-cost for the admission was allocated, even if part of the admission was in an earlier period. Only when a cost could be allocated to a specific period,were the complications included in that period.

For outpatients we used the action diagnosis from the visit since using all diagnosis types would cover the whole period for an outpatient (could be several years), and thus not realisticallybe allocated to the time of the complication.

We identified the following complications:

- Ileus, ICD10=K56*
- Colectomy, SKS=KJFB*
- Toxic megacolon ICD10=K593
- Bowel perforation ICD10=K631
- Thromboembolic episodes ICD10=DI8
- Septicaemia ICD10=A4
- Hypotension, ICD10=I95
- Shock, ICD10=R571, R572
- Renal failure, ICD10= N17, N18, N19
- Dehydration ICD10=E869
- Isolation regimes, SKS=BPX

Since more than one complication may occur during an admission, we added four groups of complications to admissions that included more than one of the listed complications. The four groups were constructed from the pattern of complications in the data.

The four added groups were:

- Septicaemia and Renal failure
- Septicaemia and one other complication
- Renal failure and one other complication
- All other complication combinations

Supplementary Table 2. Number and share of COHA and HOHA patients with complications^a^

|  | **COHA** | | | | **HOHA** | | | |
| --- | --- | --- | --- | --- | --- | --- | --- | --- |
|  | **Year 1** | | **Year 2** | | **Year 1** | | **Year 2** | |
| **# patients, n** | 5,571 | | 3,782 | | 6,275 | | 3,730 | |
| **Patients dead** | N | % | N | % | N | % | N | % |
| # patients dead during the year | 1,789 | 32.1 | 446 | 11.8 | 2,545 | 40.6 | 605 | 16.2 |
| # patients with complications dead during the year | 936 | 16.8 | 178 | 4.7 | 1,039 | 16.6 | 263 | 7.1 |
| **Inpatient** | N | % | N | % | N | % | N | % |
| **Complications, Inpatient** |  |  |  |  |  |  |  |  |
| Septicaemia | 839 | 15.1 | 246 | 6.5 | 904 | 14.4 | 347 | 9.3 |
| Dehydration | 828 | 14.9 | 163 | 4.3 | 703 | 11.2 | 207 | 5.5 |
| Renal failure | 535 | 9.6 | 216 | 5.7 | 654 | 10.4 | 261 | 7.0 |
| Ileus | 103 | 1.8 | 28 | 0.7 | 114 | 1.8 | 48 | 1.3 |
| Hypotension | 50 | 0.9 | 13 | 0.3 | 70 | 1.1 | 27 | 0.7 |
| Shock | 46 | 0.8 | 16 | 0.4 | 64 | 1.0 | 21 | 0.6 |
| Thromboembolic episodes | - |  | - |  | - |  | - |  |
| Toxic megacolon | - |  | - |  | - |  | - |  |
| Bowel perforation | 6 | 0.1 | - |  | 6 | 0.1 | - |  |
| Colectomy | 71 | 1.3 | 15 | 0.4 | 70 | 1.1 | 21 | 0.6 |
| Isolation regimes | 189 | 3.4 | 48 | 1.3 | 219 | 3.5 | 48 | 1.3 |
| **No. of complications, Inpatient** |  |  |  |  |  |  |  |  |
| No complications | 3,565 | 64.0 | 3,198 | 84.6 | 4,197 | 66.9 | 2,979 | 79.9 |
| 1 complication | 1,471 | 26.4 | 451 | 11.9 | 1,488 | 23.7 | 557 | 14.9 |
| 2 complications | 430 | 7.7 | 110 | 2.9 | 476 | 7.6 | 161 | 4.3 |
| 3+ complications | 105 | 1.9 | 23 | 0.6 | 114 | 1.8 | 33 | 0.9 |
| **Outpatient** | N | % | N | % | N | % | N | % |
| **No. of complications, Outpatient** |  |  |  |  |  |  |  |  |
| Septicaemia | 93 | 1.7 | 34 | 0.9 | 86 | 1.4 | 35 | 0.9 |
| Dehydration | 50 | 0.9 | 22 | 0.6 | 27 | 0.4 | 14 | 0.4 |
| Renal failure | 371 | 6.7 | 243 | 6.4 | 456 | 7.3 | 258 | 6.9 |
| Ileus | 15 | 0.3 | 5 | 0.1 | 17 | 0.3 | 7 | 0.2 |
| Hypotension | 5 | 0.1 | - |  | 11 | 0.2 | - |  |
| Shock | - |  | - |  | - |  | - |  |
| Thromboembolic episodes | - |  | - |  | - |  | - |  |
| Toxic megacolon | - |  | - |  | - |  | - |  |
| Bowel perforation | - |  | - |  | - |  | - |  |
| Colectomy | - |  | - |  | - |  | - |  |
| Isolation regimes | - |  | - |  | - |  | - |  |
| **# Complications, Outpatient** |  |  |  |  |  |  |  |  |
| No complications | 5,052 | 90.7 | 3,482 | 92.1 | 5,690 | 90.7 | 3,419 | 91.7 |
| 1 complication | 494 | 8.9 | 294 | 7.8 | 569 | 9.1 | 304 | 8.2 |
| 2 complications | 25 | 0.4 | 6 | 0.2 | 16 | 0.3 | 7 | 0.2 |
| 3+ complications | - | 0.0 | - | 0.0 | - | 0.0 | - | 0.0 |
| **Inpatient and outpatient** | N | % | N | % | N | % | N | % |
| **Complications** |  |  |  |  |  |  |  |  |
| Septicaemia | 882 | 15.8 | 263 | 7.0 | 949 | 15.1 | 361 | 9.7 |
| Dehydration | 841 | 15.1 | 175 | 4.6 | 718 | 11.4 | 217 | 5.8 |
| Renal failure | 610 | 10.9 | 310 | 8.2 | 779 | 12.4 | 376 | 10.1 |
| Ileus | 104 | 1.9 | 29 | 0.8 | 120 | 1.9 | 50 | 1.3 |
| Hypotension | 54 | 1.0 | 13 | 0.3 | 79 | 1.3 | 29 | 0.8 |
| Shock | 47 | 0.8 | 16 | 0.4 | 65 | 1.0 | 21 | 0.6 |
| Thromboembolic episodes | - |  | - |  | - |  | - |  |
| Toxic megacolon | 6 | 0.1 | - |  | 5 | 0.1 | - |  |
| Bowel perforation | 6 | 0.1 | - |  | 7 | 0.1 | - |  |
| Colectomy | 71 | 1.3 | - |  | 70 | 1.1 | 21 | 0.6 |
| Isolation regimes | 189 | 3.4 | 48 | 1.3 | 219 | 3.5 | 48 | 1.3 |
| **No. of complications** |  |  |  |  |  |  |  |  |
| No complications | 3,457 | 62.1 | 3,088 | 81.6 | 4,120 | 65.7 | 2,853 | 76.5 |
| 1 complication | 1,553 | 27.9 | 548 | 14.5 | 1,612 | 25.7 | 674 | 18.1 |
| 2 complications | 452 | 8.1 | 121 | 3.2 | 508 | 8.1 | 172 | 4.6 |
| 3+ complications | 109 | 2.0 | 25 | 0.7 | 35 | 0.6 | 31 | 0.8 |

a) Due to anonymity requirements, no cells with less than five observations are listed. In such cases, distributions do not always amount to 100%

Supplementary Table 3. Average hospital days due to complications in Year 1 (excluding index admission) and Year 2 for COHA and HOHA patients ^a^

|  | **COHA** | | | | | | **HOHA** | | | | | |
| --- | --- | --- | --- | --- | --- | --- | --- | --- | --- | --- | --- | --- |
|  | **Year 1** | | | **Year 2** | | | **Year 1** | | | **Year 2** | | |
| **# patients, n** | 5,571 | | | 3,782 | | | 6,275 | | | 3,730 | | |
|  | Hospitalized days per year | | Share of Hospitalized days total | Hospitalized days per year | | Share of Hospitalized days total | Hospitalized days per year | | Share of Hospitalized days total | Hospitalized days per year | | Share of Hospitalized days total |
|  | Mean | Std. | % | Mean | Std. | % | Mean | Std. | % | Mean | Std. | % |
| **Hospitalized days total** | 17.7 | 23.9 |  | 8.0 | 17.1 |  | 21.7 | 31.6 |  | 10.5 | 20.1 |  |
| **Hospitalized days due to complications** | 6.1 | 15.1 | 34.6 | 2.6 | 10.8 | 32.2 | 7.1 | 19.7 | 32.6 | 3.4 | 11.3 | 32.4 |
| **Complications hospitalized days** |  |  |  |  |  |  |  |  |  |  |  |  |
| Septicaemia | 1.4 | 6.1 | 8.0 | 0.6 | 4.0 | 7.9 | 1.8 | 8.4 | 8.2 | 0.9 | 4.6 | 8.9 |
| Dehydration | 1.3 | 5.3 | 7.6 | 0.3 | 3.0 | 3.9 | 0.9 | 4.6 | 4.3 | 0.3 | 2.3 | 3.2 |
| Renal failure | 1.3 | 6.6 | 7.4 | 0.8 | 5.6 | 9.7 | 1.6 | 9.2 | 7.4 | 0.9 | 6.1 | 9.0 |
| Ileus | 0.1 | 1.9 | 0.8 | 0.1 | 1.4 | 1.0 | 0.2 | 3.1 | 0.9 | 0.1 | 1.7 | 1.1 |
| Hypotension | 0.1 | 1.3 | 0.3 | 0.0 | 0.8 | 0.3 | 0.1 | 1.4 | 0.3 | 0.0 | 0.4 | 0.2 |
| Shock | 0.0 | 0.6 | 0.1 | 0.0 | 0.6 | 0.2 | 0.0 | 1.3 | 0.2 | 0.0 | 1.1 | 0.2 |
| Thromboembolic episodes | - | - |  | - | - |  | - | - |  | - | - |  |
| Toxic megacolon | - | - |  | - | - |  | - | - |  | - | - |  |
| Bowel perforation | 0.0 | 0.2 | 0.0 | - | - |  | - | - |  | - | - |  |
| Colectomy | 0.2 | 2.9 | 0.9 | 0.0 | 0.9 | 0.5 | 0.1 | 1.8 | 0.4 | 0.0 | 0.8 | 0.4 |
| Isolation regimes | 0.4 | 4.5 | 2.2 | 0.2 | 4.7 | 3.1 | 0.6 | 6.7 | 2.8 | 0.2 | 3.4 | 2.2 |
| Mixed complications |  |  |  |  |  |  |  |  |  |  |  |  |
| Septicaemia and Renal failure | 0.5 | 5.8 | 2.9 | 0.2 | 2.7 | 2.4 | 0.6 | 6.5 | 2.9 | 0.3 | 3.3 | 2.9 |
| Septicaemia and other complication | 0.4 | 4.3 | 2.5 | 0.1 | 2.2 | 1.4 | 0.6 | 7.3 | 2.7 | 0.2 | 2.4 | 1.5 |
| Renal failure andother complication | 0.2 | 2.4 | 1.0 | 0.1 | 2.0 | 1.1 | 0.3 | 5.0 | 1.4 | 0.2 | 3.2 | 2.0 |
| All other complication combinations | 0.2 | 2.3 | 0.9 | 0.1 | 1.9 | 0.7 | 0.2 | 3.4 | 0.8 | 0.1 | 1.4 | 0.7 |

a) Due to anonymity requirements, no cells with less than five observations are listed. In such cases, distributions do not always amount to 100%

Supplementary Table 4.1. Average inpatient (hospitalised) cost due to complications in Year 1 (excl. index cost) and Year 2,for COHA and HOHA patients ^a^

|  | **COHA** | | | | | | **HOHA** | | | | | |
| --- | --- | --- | --- | --- | --- | --- | --- | --- | --- | --- | --- | --- |
|  | **Year 1** | | | **Year 2** | | | **Year 1** | | | **Year 2** | | |
| **# patients, n** | 5,571 | | | 3,782 | | | 6,275 | | | 3,730 | | |
|  | Inpatient cost (Hospitalized) | | Share of total inpatient cost | Inpatient cost (Hospitalized) | | Share of total inpatient cost | Inpatient cost (Hospitalized) | | Share of total inpatient cost | Inpatient cost (Hospitalized) | | Share of total inpatient cost |
|  | Mean (€) | Std. | % | Mean (€) | Std. | % | Mean (€) | Std. | % | Mean (€) | Std. | % |
| **Total inpatient cost** | 16,369 | 34,038 |  | 9,002 | 25,399 |  | 20,793 | 41,753 |  | 11,702 | 32,269 |  |
| **Inpatient cost due to complications** | 5,931 | 19,102 | 36.2 | 2,935 | 16,064 | 32.6 | 7,345 | 22,375 | 35.3 | 3,783 | 15,827 | 32.3 |
| **Complications inpatient cost** |  |  |  |  |  |  |  |  |  |  |  |  |
| Septicaemia | 1,187 | 5,193 | 7.3 | 523 | 3,426 | 5.8 | 1,560 | 7,474 | 7.5 | 890 | 5,470 | 7.6 |
| Dehydration | 774 | 2,931 | 4.7 | 257 | 2,017 | 2.9 | 669 | 3,198 | 3.2 | 259 | 1,526 | 2.2 |
| Renal failure | 1,486 | 7,741 | 9.1 | 833 | 5,599 | 9.3 | 1,546 | 8,105 | 7.4 | 931 | 5,655 | 8.0 |
| Ileus | 112 | 1,337 | 0.7 | 69 | 1,512 | 0.8 | 143 | 2,233 | 0.7 | 164 | 3,860 | 1.4 |
| Hypotension | 42 | 811 | 0.3 | 17 | 516 | 0.2 | 55 | 1,092 | 0.3 | 27 | 523 | 0.2 |
| Shock | 34 | 834 | 0.2 | 56 | 2,038 | 0.6 | 39 | 1,114 | 0.2 | 40 | 1,511 | 0.3 |
| Thromboembolic episodes | - | - |  | - | - |  | - | - |  | - | - |  |
| Toxic megacolon | - | - |  | - | - |  | - | - |  | - | - |  |
| Bowel perforation | 5 | 250 | 0.0 | - | - |  | - | - |  | - | - |  |
| Colectomy | 135 | 1,652 | 0.8 | 42 | 843 | 0.5 | 88 | 1,487 | 0.4 | 51 | 961 | 0.4 |
| Isolation regimes | 800 | 12,320 | 4.9 | 503 | 10,984 | 5.6 | 1,141 | 12,444 | 5.5 | 529 | 8,182 | 4.5 |
| Mixed complications |  |  |  |  |  |  |  |  |  |  |  |  |
| Septicaemia and Renal failure | 620 | 5,769 | 3.8 | 254 | 3,171 | 2.8 | 810 | 8,539 | 3.9 | 366 | 4,066 | 3.1 |
| Septicaemia and other complication | 441 | 4,447 | 2.7 | 153 | 4,073 | 1.7 | 685 | 7,526 | 3.3 | 245 | 4,208 | 2.1 |
| Renal failure and other complication | 151 | 1,891 | 0.9 | 180 | 4,434 | 2.0 | 364 | 4,826 | 1.8 | 219 | 4,178 | 1.9 |
| All other complication combinations | 138 | 2,002 | 0.8 | 47 | 1,450 | 0.5 | 188 | 2,827 | 0.9 | 59 | 1,357 | 0.5 |

a) Due to anonymity requirements, no cells with less than five observations are listed. In such cases, distributions do not always amount to 100%

Supplementary Table 4.2. Average outpatientcost due to complications in Year 1 (excl. index cost) and Year 2,for COHA and HOHA patients ^a^

|  | **COHA** | | | | | | **HOHA** | | | | | |
| --- | --- | --- | --- | --- | --- | --- | --- | --- | --- | --- | --- | --- |
|  | **Year 1** | | | **Year 2** | | | **Year 1** | | | **Year 2** | | |
| **# patients, n** | 5,571 | | | 3,782 | | | 6,275 | | | 3,730 | | |
|  | Outpatient cost | | Share of total outpatient cost | Outpatient cost | | Share of total outpatient cost | Outpatient cost | | Share of total outpatient cost | Outpatient cost | | Share of total outpatient cost |
|  | Mean (€) | Std. | % | Mean (€) | Std. | % | Mean (€) | Std. | % | Mean (€) | Std. | % |
| **Total outpatient cost** | 6,669 | 22,695 |  | 5,600 | 14,530 |  | 4,998 | 12,587 |  | 4,695 | 12,533 |  |
| **Outpatient cost due to complications** | 1,713 | 9,717 | 25.7 | 1,748 | 9,837 | 31.2 | 1,256 | 8,039 | 25.1 | 1,361 | 8,265 | 29.0 |
| **Complications outpatient cost** |  |  |  |  |  |  |  |  |  |  |  |  |
| Septicaemia | 45 | 1,438 | 0.7 | 44 | 1,566 | 0.8 | 33 | 1,117 | 0.7 | 22 | 644 | 0.5 |
| Dehydration | 10 | 280 | 0.1 | 7 | 275 | 0.1 | 2 | 63 | 0.0 | 2 | 63 | 0.0 |
| Renal failure | 1,656 | 9,578 | 24.8 | 1,697 | 9,696 | 30.3 | 1,219 | 7,924 | 24.4 | 1,336 | 8,199 | 28.5 |
| Ileus | 1 | 45 | 0.0 | 0 | 8 | 0.0 | 1 | 19 | 0.0 | 1 | 23 | 0.0 |
| Hypotension | 0 | 21 | 0.0 | - | - |  | 1 | 22 | 0.0 | - | - | 0.0 |
| Shock | - | - |  | - | - |  | - | - |  | - | - |  |
| Thromboembolic episodes | - | - |  | - | - |  | - | - |  | - | - |  |
| Toxic megacolon | - | - |  | - | - |  | - | - |  | - | - |  |
| Bowel perforation | - | - |  | - | - |  | - | - |  | - | - |  |
| Colectomy | - | - |  | - | - |  | - | - |  | - | - |  |
| Isolation regimes | - | - |  | - | - |  | - | - |  | - | - |  |

a) Due to anonymity requirements, no cells with less than five observations are listed. In such cases, distributions do not always amount to 100%

### Cost analysis

Cost from DRG and DAGS were prices including rehabilitation.Index admission costs for HOHA were from the Cost Database, not the DRG Register.

The cost analysis (Supplementary Tables 5.1-5.9) were split into types of health cost:

- Prescription medication
- Primary sector
- Somatic inpatient
- Somatic outpatient
- Psychiatric inpatient
- Psychiatric outpatient
- All health cost (sum of the above mentioned)

Confidence intervals for the cost analysis can be found in the Supplementary Tables 5.4-5.9. For transfer payment, we calculated the number of months, since we did not have a price for these transfers. Only transfers for people in the workforce were in the data, thus age pension wasunavailable.

The first analysis included average cost and a test for significant difference between case and controls. Since cases and controls were matched, we used a bootstrapped t-test and thus had no additional explanatory variables included in the model.

We looked at the sub-periods:

- 12 month pre-period (12 months)
- Index admission(HOHA, cost from Cost Database)/index visit/index medico/index primary
- 1-3 months post-period (3 months)
- 4-6 months post-period (3 months)
- 7-9 months post-period (3 months)
- 10-12 months post-period (3 months)
- 13-24 months post-period (12 months)

Patients were included, if they were alive at least part of the sub-period. That is, they were included even if they died during the period. The number of patients was thus declining in the sub-periods, since they were excluded in the periods following the period were death occurred. The average cost wastherefore a measure for treatment cost in each period and each group.

Supplementary Table 5.1. CDI health care costs for CDI

|  |  | | **Health cost CDI** | | | | | | | | | | | | | | | | | | | | |
| --- | --- | --- | --- | --- | --- | --- | --- | --- | --- | --- | --- | --- | --- | --- | --- | --- | --- | --- | --- | --- | --- | --- | --- |
|  | **# patients** | | **Somatic inpatient** | | | **Somatic outpatient** | | | **Psychiatric inpatient** | | | **Psychiatric outpatient** | | | **Primary health sector** | | | **Prescription Drugs** | | | **Total health cost** | | |
|  | **CDI** | **Control** | **CDI** | **Control** | **P-value** | **CDI** | **Control** | **P-value** | **CDI** | **Control** | **P-value** | **CDI** | **Control** | **P-value** | **CDI** | **Control** | **P-value** | **CDI** | **Control** | **P-value** | **CDI** | **Control** | **P-value** |
| **Period** | **N** | **N** | **€** | **€** |  | **€** | **€** |  | **€** | **€** |  | **€** | **€** |  | **€** | **€** |  | **€** | **€** |  | **€** | **€** |  |
| Year -1 | 12,768 | 23,272 | 22,160 | 10,035 | <0.001 | 6,203 | 4,365 | <0.001 | 201 | 155 | 0.627 | 58 | 51 | 0.886 | 705 | 662 | <0.001 | 1,168 | 992 | <0.001 | 30,494 | 16,261 | <0.001 |
| Index cost** | 12,768 | 23,272 | 12,817 | 4,456 | <0.001 | 24 | 44 | <0.001 | 1 | 1 | 0.994 | 0 | 0 | 1.000 | 12 | 15 | <0.001 | 12 | 7 | <0.001 | 12,867 | 4,522 | <0.001 |
| Month 1-3 | 11,846 | 22,134 | 9,510 | 5,410 | <0.001 | 1,963 | 1,588 | <0.001 | 76 | 45 | 0.106 | 15 | 15 | 1.000 | 195 | 166 | <0.001 | 343 | 273 | <0.001 | 12,102 | 7,498 | <0.001 |
| Month 4-6 | 9,196 | 19,873 | 5,197 | 2,929 | <0.001 | 1,943 | 1,301 | <0.001 | 67 | 40 | 0.435 | 19 | 17 | 0.913 | 193 | 169 | <0.001 | 323 | 260 | <0.001 | 7,742 | 4,716 | <0.001 |
| Month 7-9 | 8,366 | 18,901 | 3,876 | 2,241 | <0.001 | 1,744 | 1,101 | <0.001 | 56 | 49 | 0.998 | 18 | 16 | 0.949 | 191 | 166 | <0.001 | 316 | 254 | <0.001 | 6,200 | 3,826 | <0.001 |
| Month 10-12 | 7,883 | 18,184 | 3,158 | 2,014 | <0.001 | 1,624 | 973 | <0.001 | 41 | 51 | 0.997 | 18 | 17 | 0.997 | 191 | 164 | <0.001 | 312 | 253 | <0.001 | 5,344 | 3,472 | <0.001 |
| Year 2 | 7,512 | 17,580 | 10,342 | 6,089 | <0.001 | 5,151 | 3,442 | <0.001 | 182 | 129 | 0.563 | 76 | 61 | 0.528 | 717 | 628 | <0.001 | 1,122 | 910 | <0.001 | 17,590 | 11,260 | <0.001 |

*P-value from t-test and bootstrapped; **Index admissions are calculated from the cost database for HOHA. Since COHA is partly outpatient onset, their index admission is from DRG

Supplementary Table 5.2. CDI health care costs for COHA

|  |  | | **Health cost COHA** | | | | | | | | | | | | | | | | | | | | |
| --- | --- | --- | --- | --- | --- | --- | --- | --- | --- | --- | --- | --- | --- | --- | --- | --- | --- | --- | --- | --- | --- | --- | --- |
|  | **# patients** | | **Somatic inpatient** | | | **Somatic outpatient** | | | **Psychiatric inpatient** | | | **Psychiatric outpatient** | | | **Primary health sector** | | | **Prescription Drugs** | | | **Total health cost** | | |
|  | **COHA** | **Control** | **COHA** | **Control** | **P-value** | **COHA** | **Control** | **P-value** | **COHA** | **Control** | **P-value** | **COHA** | **Control** | **P-value** | **COHA** | **Control** | **P-value** | **COHA** | **Control** | **P-value** | **COHA** | **Control** | **P-value** |
| **Period** | **N** | **N** | **€** | **€** |  | **€** | **€** |  | **€** | **€** |  | **€** | **€** |  | **€** | **€** |  | **€** | **€** |  | **€** | **€** |  |
| Year -1 | 5,585 | 10,204 | 20,002 | 7,014 | <0.001 | 7,430 | 4,122 | <0.001 | 217 | 179 | 0.981 | 66 | 60 | 0.998 | 731 | 623 | <0.001 | 1,167 | 876 | <0.001 | 29,613 | 12,873 | <0.001 |
| Index cost** | 5,585 | 10,204 | 3,624 | 2,881 | <0.001 | 51 | 95 | <0.001 | 1 | 1 | 1.000 | 0 | 0 | 0.998 | 18 | 13 | <0.001 | 10 | 6 | <0.001 | 3,704 | 2,996 | <0.001 |
| Month 1-3 | 5,571 | 9,868 | 8,354 | 4,368 | <0.001 | 2,229 | 1,438 | <0.001 | 107 | 51 | 0.058 | 17 | 17 | 1.000 | 212 | 155 | <0.001 | 325 | 238 | <0.001 | 11,244 | 6,267 | <0.001 |
| Month 4-6 | 4,502 | 9,092 | 4,422 | 2,282 | <0.001 | 2,149 | 1,158 | <0.001 | 90 | 47 | 0.594 | 21 | 18 | 0.996 | 190 | 155 | <0.001 | 314 | 227 | <0.001 | 7,187 | 3,887 | <0.001 |
| Month 7-9 | 4,140 | 8,768 | 3,381 | 1,748 | <0.001 | 1,941 | 1,022 | <0.001 | 36 | 41 | 1.000 | 20 | 17 | 0.978 | 184 | 153 | <0.001 | 299 | 221 | <0.001 | 5,861 | 3,203 | <0.001 |
| Month 10-12 | 3,938 | 8,515 | 2,729 | 1,578 | <0.001 | 1,783 | 898 | <0.001 | 55 | 67 | 0.999 | 21 | 19 | 0.999 | 183 | 152 | <0.001 | 309 | 214 | <0.001 | 5,081 | 2,928 | <0.001 |
| Year 2 | 3,782 | 8,305 | 9,002 | 4,980 | <0.001 | 5,600 | 3,273 | <0.001 | 187 | 136 | 0.914 | 89 | 67 | 0.653 | 680 | 580 | <0.001 | 1,082 | 814 | <0.001 | 16,640 | 9,850 | <0.001 |

*P-value from t-test and bootstrapped; **Index admissions are calculated from the cost database for HOHA. Since COHA is partly outpatient onset, their index admission is from DRG

Supplementary Table 5.3. CDI health care costs for HOHA

|  |  | | **Health cost HOHA** | | | | | | | | | | | | | | | | | | | | |
| --- | --- | --- | --- | --- | --- | --- | --- | --- | --- | --- | --- | --- | --- | --- | --- | --- | --- | --- | --- | --- | --- | --- | --- |
|  | **# patients** | | **Somatic inpatient** | | | **Somatic outpatient** | | | **Psychiatric inpatient** | | | **Psychiatric outpatient** | | | **Primary health sector** | | | **Prescription Drugs** | | | **Total health cost** | | |
|  | **HOHA** | **Control** | **HOHA** | **Control** | **P-value** | **HOHA** | **Control** | **P-value** | **HOHA** | **Control** | **P-value** | **HOHA** | **Control** | **P-value** | **HOHA** | **Control** | **P-value** | **HOHA** | **Control** | **P-value** | **HOHA** | **Control** | **P-value** |
| **Period** | **N** | **N** | **€** | **€** |  | **€** | **€** |  | **€** | **€** |  | **€** | **€** |  | **€** | **€** |  | **€** | **€** |  | **€** | **€** |  |
| Year -1 | 7,183 | 13,068 | 23,838 | 12,394 | <0.001 | 5,249 | 4,555 | <0.001 | 188 | 136 | 0.674 | 51 | 44 | 0.868 | 684 | 693 | 0.960 | 1,169 | 1,083 | 0.044 | 31,180 | 18,906 | <0.001 |
| Index cost** | 7,183 | 13,068 | 19,965 | 5,685 | <0.001 | 4 | 3 | <0.001 | 1 | 0 | 0.878 | 0 | 0 | 0.954 | 8 | 16 | <0.001 | 14 | 9 | <0.001 | 19,992 | 5,714 | <0.001 |
| Month 1-3 | 6,275 | 12,266 | 10,537 | 6,248 | <0.001 | 1,727 | 1,708 | <0.001 | 48 | 41 | 0.999 | 13 | 14 | 1.000 | 180 | 175 | 0.488 | 360 | 301 | <0.001 | 12,864 | 8,487 | <0.001 |
| Month 4-6 | 4,694 | 10,781 | 5,941 | 3,476 | <0.001 | 1,746 | 1,422 | <0.001 | 44 | 35 | 0.989 | 17 | 15 | 0.931 | 195 | 181 | 0.002 | 330 | 287 | <0.001 | 8,274 | 5,416 | <0.001 |
| Month 7-9 | 4,226 | 10,133 | 4,360 | 2,666 | <0.001 | 1,551 | 1,169 | <0.001 | 75 | 55 | 0.981 | 16 | 15 | 0.999 | 198 | 177 | <0.001 | 332 | 283 | 0.001 | 6,533 | 4,366 | <0.001 |
| Month 10-12 | 3,945 | 9,669 | 3,586 | 2,397 | <0.001 | 1,465 | 1,040 | <0.001 | 28 | 36 | 0.999 | 16 | 15 | 1.000 | 199 | 176 | <0.001 | 314 | 287 | 0.643 | 5,607 | 3,951 | <0.001 |
| Year 2 | 3,730 | 9,275 | 11,702 | 7,082 | <0.001 | 4,695 | 3,594 | <0.001 | 177 | 123 | 0.802 | 62 | 56 | 0.992 | 756 | 672 | <0.001 | 1,161 | 996 | <0.001 | 18,553 | 12,523 | <0.001 |

*P-value from t-test and bootstrapped; **Index admissions are calculated from the cost database for HOHA. Since COHA is partly outpatient onset, their index admission is from DRG

Supplementary Table 5.4. Confidence intervals of all health care costs calculations for CDI patients(Supplementary Tables 5.1)

|  |  | **# patients** | **Somatic inpatient** | | | **Somatic outpatient** | | | **Psychiatric inpatient** | | | **Psychiatric outpatient** | | | **Primary health sector** | | | **Prescription Drugs** | | | **Total health cost** | | |
| --- | --- | --- | --- | --- | --- | --- | --- | --- | --- | --- | --- | --- | --- | --- | --- | --- | --- | --- | --- | --- | --- | --- | --- |
|  |  |  | **Mean** | **LCL** | **UCL** | **Mean** | **LCL** | **UCL** | **Mean** | **LCL** | **UCL** | **Mean** | **LCL** | **UCL** | **Mean** | **LCL** | **UCL** | **Mean** | **LCL** | **UCL** | **Mean** | **LCL** | **UCL** |
|  |  |  | **€** | **€** | **€** | **€** | **€** | **€** | **€** | **€** | **€** | **€** | **€** | **€** | **€** | **€** | **€** | **€** | **€** | **€** | **€** | **€** | **€** |
| Year -1 (12 month before index) | | 12,768 | 22,160 | 21,468 | 22,852 | 6,203 | 5,966 | 6,440 | 201 | 147 | 254 | 58 | 47 | 68 | 705 | 692 | 718 | 1,168 | 1,132 | 1,204 | 30,494 | 29,727 | 31,262 |
| Index cost** | | 12,768 | 12,817 | 12,406 | 13,228 | 24 | 22 | 27 | 1 | 0 | 3 | 0 | 0 | 0 | 12 | 11 | 13 | 12 | 11 | 13 | 12,867 | 12,457 | 13,277 |
| Month 1-3 | | 11,846 | 9,510 | 9,149 | 9,871 | 1,963 | 1,871 | 2,055 | 76 | 52 | 100 | 15 | 12 | 17 | 195 | 191 | 199 | 343 | 333 | 354 | 12,102 | 11,723 | 12,481 |
| Month 4-6 | | 9,196 | 5,197 | 4,892 | 5,503 | 1,943 | 1,837 | 2,049 | 67 | 36 | 98 | 19 | 15 | 23 | 193 | 188 | 198 | 323 | 310 | 335 | 7,742 | 7,403 | 8,081 |
| Month 7-9 | | 8,366 | 3,876 | 3,594 | 4,158 | 1,744 | 1,605 | 1,883 | 56 | 29 | 84 | 18 | 14 | 22 | 191 | 185 | 196 | 316 | 302 | 329 | 6,200 | 5,872 | 6,528 |
| Month 10-12 | | 7,883 | 3,158 | 2,904 | 3,411 | 1,624 | 1,480 | 1,768 | 41 | 20 | 63 | 18 | 14 | 23 | 191 | 185 | 197 | 312 | 298 | 325 | 5,344 | 5,040 | 5,648 |
| Year 2 (month 13-24) | | 7,512 | 10,342 | 9,686 | 10,999 | 5,151 | 4,843 | 5,458 | 182 | 120 | 244 | 76 | 60 | 91 | 717 | 696 | 739 | 1,122 | 1,072 | 1,172 | 17,590 | 16,799 | 18,381 |

Supplementary Table 5.5. Confidence intervals of all health care costs calculations for all controls (Supplementary Tables 5.1)

|  |  | **# patients** | **Somatic inpatient** | | | **Somatic outpatient** | | | **Psychiatric inpatient** | | | **Psychiatric outpatient** | | | **Primary health sector** | | | **Prescription Drugs** | | | **Total health cost** | | |
| --- | --- | --- | --- | --- | --- | --- | --- | --- | --- | --- | --- | --- | --- | --- | --- | --- | --- | --- | --- | --- | --- | --- | --- |
|  |  |  | **Mean** | **LCL** | **UCL** | **Mean** | **LCL** | **UCL** | **Mean** | **LCL** | **UCL** | **Mean** | **LCL** | **UCL** | **Mean** | **LCL** | **UCL** | **Mean** | **LCL** | **UCL** | **Mean** | **LCL** | **UCL** |
|  |  |  | **€** | **€** | **€** | **€** | **€** | **€** | **€** | **€** | **€** | **€** | **€** | **€** | **€** | **€** | **€** | **€** | **€** | **€** | **€** | **€** | **€** |
| Year -1 (12 month before index) | | 23,272 | 10,035 | 9,715 | 10,355 | 4,365 | 4,232 | 4,498 | 155 | 119 | 191 | 51 | 45 | 58 | 662 | 653 | 671 | 992 | 964 | 1,020 | 16,261 | 15,883 | 16,638 |
| Index cost** | | 23,272 | 4,456 | 4,339 | 4,572 | 44 | 41 | 46 | 1 | 0 | 2 | 0 | 0 | 0 | 15 | 14 | 15 | 7 | 7 | 8 | 4,522 | 4,406 | 4,638 |
| Month 1-3 | | 22,134 | 5,410 | 5,233 | 5,587 | 1,588 | 1,531 | 1,645 | 45 | 32 | 59 | 15 | 13 | 18 | 166 | 164 | 169 | 273 | 265 | 281 | 7,498 | 7,303 | 7,692 |
| Month 4-6 | | 19,873 | 2,929 | 2,788 | 3,071 | 1,301 | 1,254 | 1,349 | 40 | 25 | 55 | 17 | 14 | 19 | 169 | 166 | 172 | 260 | 252 | 267 | 4,716 | 4,559 | 4,874 |
| Month 7-9 | | 18,901 | 2,241 | 2,120 | 2,361 | 1,101 | 1,056 | 1,147 | 49 | 30 | 67 | 16 | 13 | 18 | 166 | 163 | 169 | 254 | 244 | 265 | 3,826 | 3,689 | 3,964 |
| Month 10-12 | | 18,184 | 2,014 | 1,895 | 2,132 | 973 | 933 | 1,014 | 51 | 29 | 72 | 17 | 14 | 20 | 164 | 161 | 168 | 253 | 239 | 267 | 3,472 | 3,338 | 3,606 |
| Year 2 (month 13-24) | | 17,580 | 6,089 | 5,859 | 6,318 | 3,442 | 3,301 | 3,584 | 129 | 94 | 165 | 61 | 52 | 71 | 628 | 617 | 640 | 910 | 878 | 942 | 11,260 | 10,957 | 11,564 |

Supplementary Table 5.6. Confidence intervals of all health care costs calculations for COHA patients (Supplementary Tables 5.2)

|  |  | **# patients** | **Somatic inpatient** | | | **Somatic outpatient** | | | **Psychiatric inpatient** | | | **Psychiatric outpatient** | | | **Primary health sector** | | | **Prescription Drugs** | | | **Total health cost** | | |
| --- | --- | --- | --- | --- | --- | --- | --- | --- | --- | --- | --- | --- | --- | --- | --- | --- | --- | --- | --- | --- | --- | --- | --- |
|  |  |  | **Mean** | **LCL** | **UCL** | **Mean** | **LCL** | **UCL** | **Mean** | **LCL** | **UCL** | **Mean** | **LCL** | **UCL** | **Mean** | **LCL** | **UCL** | **Mean** | **LCL** | **UCL** | **Mean** | **LCL** | **UCL** |
|  |  |  | **€** | **€** | **€** | **€** | **€** | **€** | **€** | **€** | **€** | **€** | **€** | **€** | **€** | **€** | **€** | **€** | **€** | **€** | **€** | **€** | **€** |
| Year -1 (12 month before index) | | 5,585 | 20,002 | 19,006 | 20,999 | 7,430 | 7,014 | 7,846 | 217 | 130 | 304 | 66 | 47 | 84 | 731 | 711 | 752 | 1,167 | 1,114 | 1,220 | 29,613 | 28,473 | 30,753 |
| Index cost** | | 5,585 | 3,624 | 3,444 | 3,803 | 51 | 45 | 57 | 1 | 0 | 3 | 0 | 0 | 1 | 18 | 16 | 19 | 10 | 9 | 11 | 3,704 | 3,525 | 3,882 |
| Month 1-3 | | 5,571 | 8,354 | 7,917 | 8,791 | 2,229 | 2,073 | 2,385 | 107 | 67 | 147 | 17 | 13 | 21 | 212 | 205 | 218 | 325 | 310 | 340 | 11,244 | 10,768 | 11,720 |
| Month 4-6 | | 4,502 | 4,422 | 4,056 | 4,788 | 2,149 | 1,972 | 2,326 | 90 | 32 | 149 | 21 | 14 | 27 | 190 | 183 | 198 | 314 | 295 | 333 | 7,187 | 6,755 | 7,619 |
| Month 7-9 | | 4,140 | 3,381 | 2,978 | 3,785 | 1,941 | 1,691 | 2,190 | 36 | 12 | 61 | 20 | 14 | 25 | 184 | 176 | 191 | 299 | 280 | 318 | 5,861 | 5,367 | 6,355 |
| Month 10-12 | | 3,938 | 2,729 | 2,435 | 3,023 | 1,783 | 1,529 | 2,038 | 55 | 16 | 95 | 21 | 14 | 28 | 183 | 176 | 191 | 309 | 287 | 331 | 5,081 | 4,674 | 5,488 |
| Year 2 (month 13-24) | | 3,782 | 9,002 | 8,192 | 9,812 | 5,600 | 5,137 | 6,063 | 187 | 106 | 267 | 89 | 62 | 116 | 680 | 652 | 707 | 1,082 | 1,013 | 1,152 | 16,640 | 15,606 | 17,674 |

Supplementary Table 5.7. Confidence intervals of all health care costs calculations for COHA matched controls (Supplementary Tables 5.2)

|  |  | **# patients** | **Somatic inpatient** | | | **Somatic outpatient** | | | **Psychiatric inpatient** | | | **Psychiatric outpatient** | | | **Primary health sector** | | | **Prescription Drugs** | | | **Total health cost** | | |
| --- | --- | --- | --- | --- | --- | --- | --- | --- | --- | --- | --- | --- | --- | --- | --- | --- | --- | --- | --- | --- | --- | --- | --- |
|  |  |  | **Mean** | **LCL** | **UCL** | **Mean** | **LCL** | **UCL** | **Mean** | **LCL** | **UCL** | **Mean** | **LCL** | **UCL** | **Mean** | **LCL** | **UCL** | **Mean** | **LCL** | **UCL** | **Mean** | **LCL** | **UCL** |
|  |  |  | **€** | **€** | **€** | **€** | **€** | **€** | **€** | **€** | **€** | **€** | **€** | **€** | **€** | **€** | **€** | **€** | **€** | **€** | **€** | **€** | **€** |
| Year -1 (12 month before index) | | 10,204 | 7,014 | 6,619 | 7,408 | 4,122 | 3,926 | 4,317 | 179 | 117 | 241 | 60 | 48 | 72 | 623 | 610 | 636 | 876 | 835 | 917 | 12,873 | 12,388 | 13,358 |
| Index cost** | | 10,204 | 2,881 | 2,774 | 2,987 | 95 | 89 | 101 | 1 | 1 | 3 | 0 | 0 | 0 | 13 | 12 | 14 | 6 | 5 | 6 | 2,996 | 2,890 | 3,101 |
| Month 1-3 | | 9,868 | 4,368 | 4,142 | 4,594 | 1,438 | 1,364 | 1,512 | 51 | 28 | 74 | 17 | 13 | 22 | 155 | 152 | 159 | 238 | 227 | 249 | 6,267 | 6,017 | 6,517 |
| Month 4-6 | | 9,092 | 2,282 | 2,112 | 2,451 | 1,158 | 1,092 | 1,224 | 47 | 20 | 74 | 18 | 13 | 24 | 155 | 151 | 160 | 227 | 215 | 238 | 3,887 | 3,691 | 4,082 |
| Month 7-9 | | 8,768 | 1,748 | 1,574 | 1,923 | 1,022 | 958 | 1,086 | 41 | 22 | 60 | 17 | 12 | 22 | 153 | 149 | 157 | 221 | 206 | 235 | 3,203 | 3,003 | 3,402 |
| Month 10-12 | | 8,515 | 1,578 | 1,424 | 1,732 | 898 | 841 | 955 | 67 | 31 | 104 | 19 | 14 | 24 | 152 | 147 | 156 | 214 | 200 | 227 | 2,928 | 2,749 | 3,106 |
| Year 2 (month 13-24) | | 8,305 | 4,980 | 4,697 | 5,263 | 3,273 | 3,079 | 3,467 | 136 | 78 | 195 | 67 | 51 | 84 | 580 | 565 | 594 | 814 | 761 | 867 | 9,850 | 9,450 | 10,250 |

Supplementary Table 5.8. Confidence intervals of all health care costs calculations for HOHA patients (Supplementary Tables 5.3)

|  |  | **# patients** | **Somatic inpatient** | | | **Somatic outpatient** | | | **Psychiatric inpatient** | | | **Psychiatric outpatient** | | | **Primary health sector** | | | **Prescription Drugs** | | | **Total health cost** | | |
| --- | --- | --- | --- | --- | --- | --- | --- | --- | --- | --- | --- | --- | --- | --- | --- | --- | --- | --- | --- | --- | --- | --- | --- |
|  |  |  | **Mean** | **LCL** | **UCL** | **Mean** | **LCL** | **UCL** | **Mean** | **LCL** | **UCL** | **Mean** | **LCL** | **UCL** | **Mean** | **LCL** | **UCL** | **Mean** | **LCL** | **UCL** | **Mean** | **LCL** | **UCL** |
|  |  |  | **€** | **€** | **€** | **€** | **€** | **€** | **€** | **€** | **€** | **€** | **€** | **€** | **€** | **€** | **€** | **€** | **€** | **€** | **€** | **€** | **€** |
| Year -1 (12 month before index) | | 7,183 | 23,838 | 22,883 | 24,792 | 5,249 | 4,981 | 5,517 | 188 | 122 | 255 | 51 | 39 | 63 | 684 | 667 | 701 | 1,169 | 1,121 | 1,217 | 31,180 | 30,142 | 32,217 |
| Index cost** | | 7,183 | 19,965 | 19,294 | 20,637 | 4 | 2 | 6 | 1 | 1 | 3 | 0 | 0 | 0 | 8 | 7 | 8 | 14 | 13 | 15 | 19,992 | 19,321 | 20,663 |
| Month 1-3 | | 6,275 | 10,537 | 9,977 | 11,097 | 1,727 | 1,623 | 1,831 | 48 | 20 | 76 | 13 | 10 | 16 | 180 | 175 | 185 | 360 | 345 | 375 | 12,864 | 12,288 | 13,440 |
| Month 4-6 | | 4,694 | 5,941 | 5,457 | 6,425 | 1,746 | 1,626 | 1,866 | 44 | 21 | 67 | 17 | 13 | 22 | 195 | 189 | 202 | 330 | 313 | 348 | 8,274 | 7,756 | 8,792 |
| Month 7-9 | | 4,226 | 4,360 | 3,966 | 4,754 | 1,551 | 1,427 | 1,676 | 75 | 26 | 124 | 16 | 11 | 21 | 198 | 190 | 206 | 332 | 313 | 351 | 6,533 | 6,100 | 6,966 |
| Month 10-12 | | 3,945 | 3,586 | 3,174 | 3,998 | 1,465 | 1,328 | 1,601 | 28 | 9 | 46 | 16 | 11 | 21 | 199 | 190 | 207 | 314 | 298 | 331 | 5,607 | 5,155 | 6,059 |
| Year 2 (month 13-24) | | 3,730 | 11,702 | 10,666 | 12,738 | 4,695 | 4,293 | 5,097 | 177 | 84 | 271 | 62 | 46 | 78 | 756 | 723 | 788 | 1,161 | 1,089 | 1,233 | 18,553 | 17,354 | 19,752 |

Supplementary Table 5.9. Confidence intervals of all health care costs calculations for HOHA matched controls (Supplementary Tables 5.3)

|  |  | **# patients** | **Somatic inpatient** | | | **Somatic outpatient** | | | **Psychiatric inpatient** | | | **Psychiatric outpatient** | | | **Primary health sector** | | | **Prescription Drugs** | | | **Total health cost** | | |
| --- | --- | --- | --- | --- | --- | --- | --- | --- | --- | --- | --- | --- | --- | --- | --- | --- | --- | --- | --- | --- | --- | --- | --- |
|  |  |  | **Mean** | **LCL** | **UCL** | **Mean** | **LCL** | **UCL** | **Mean** | **LCL** | **UCL** | **Mean** | **LCL** | **UCL** | **Mean** | **LCL** | **UCL** | **Mean** | **LCL** | **UCL** | **Mean** | **LCL** | **UCL** |
|  |  |  | **€** | **€** | **€** | **€** | **€** | **€** | **€** | **€** | **€** | **€** | **€** | **€** | **€** | **€** | **€** | **€** | **€** | **€** | **€** | **€** | **€** |
| Year -1 (12 month before index) | | 13,068 | 12,394 | 11,919 | 12,870 | 4,555 | 4,373 | 4,736 | 136 | 95 | 178 | 44 | 37 | 51 | 693 | 680 | 706 | 1,083 | 1,045 | 1,122 | 18,906 | 18,355 | 19,457 |
| Index cost** | | 13,068 | 5,685 | 5,498 | 5,873 | 3 | 2 | 4 | 0 | 0 | 1 | 0 | 0 | 0 | 16 | 15 | 17 | 9 | 7 | 10 | 5,714 | 5,526 | 5,902 |
| Month 1-3 | | 12,266 | 6,248 | 5,986 | 6,511 | 1,708 | 1,625 | 1,792 | 41 | 26 | 56 | 14 | 11 | 16 | 175 | 171 | 178 | 301 | 291 | 312 | 8,487 | 8,201 | 8,774 |
| Month 4-6 | | 10,781 | 3,476 | 3,257 | 3,694 | 1,422 | 1,355 | 1,490 | 35 | 20 | 50 | 15 | 12 | 18 | 181 | 177 | 185 | 287 | 277 | 298 | 5,416 | 5,179 | 5,654 |
| Month 7-9 | | 10,133 | 2,666 | 2,501 | 2,832 | 1,169 | 1,105 | 1,234 | 55 | 25 | 86 | 15 | 12 | 17 | 177 | 172 | 181 | 283 | 269 | 298 | 4,366 | 4,178 | 4,555 |
| Month 10-12 | | 9,669 | 2,397 | 2,221 | 2,573 | 1,040 | 983 | 1,097 | 36 | 12 | 60 | 15 | 12 | 18 | 176 | 171 | 180 | 287 | 265 | 310 | 3,951 | 3,755 | 4,147 |
| Year 2 (month 13-24) | | 9,275 | 7,082 | 6,730 | 7,434 | 3,594 | 3,390 | 3,798 | 123 | 80 | 166 | 56 | 46 | 66 | 672 | 654 | 689 | 996 | 958 | 1,034 | 12,523 | 12,074 | 12,972 |

Cost Regression

In the cost analysis above, cases and controls were matched on a number of parameters including diagnosis codes. However, since case and controls may stillhave different co-morbidity even when matched, we conducted a regression for costs controlled for the Charlson Co-morbidity Index in the 12 months pre-period.

For this cost analysis we used a 2-step gamma distributed analysis for all health costs (the sum of all types, inpatient somatic, outpatient somatic, inpatient psychiatric, outpatient psychiatric, primary sector, prescription medication). The 2-step gamma distributed analysis was selected due to 0’s in the cost variables, and the fact that data was not normally distributed, thus using an ordinary OLS model would have been inappropriate. In this model we controlled for confounders (sex and age) and comorbidity using the Charlson Co-morbidity Index in the pre-period. In the model we looked at total health costs 12 months after index (Year 1) and 13-24 months after index (Year 2).

The analysis was run for:

- CDI vs. controls
- HOHA vs. controls
- COHA vs. controls

The populations in the analysis was the following:

- All patients alive at least part of Year 1 (but not necessarily all of Year 1), this regression was run only for Year 1 cost (Table 4)
- All patients alive all of Year 1 and at least part of Year 2. This regression was run for Year 1 and Year 2 costs (Supplementary Table 5)

The reason for running the regression for different populations, was that patients who haddied during the period have less time to accumulate cost, but might be more expensive since they were probably sicker.

### Cost analysis stratified on complications

To determine to what extent patients with complications differed from patients without complications we conductedthe cost analysis for periods stratified bycomplications among cases. Complications weredefined as having at least one complication in the post-period (excluding the index-period of the admission), that is within 24 months after the index date. In example,the complication couldoccur after a given period, but the patient wouldbe allocated to the complication group for the entire period, including the pre-period.Only cases were stratifiedand the controls were their match.

Supplementary Table 7.1. CDI, Total health costs divided into patients with or without complications

|  | **Number of patients** | | | | **Total health cost** | | | | | |
| --- | --- | --- | --- | --- | --- | --- | --- | --- | --- | --- |
|  | **CDI with complications** | | **CDI no complications** | | **CDI with complications** | | | **CDI no complications** | | |
|  | **CDI - complications** | **Control** | **CDI - no complications** | **Control** | **CDI - complications** | **Control** | **P-value** | **CDI - no complications** | **Control** | **P-value** |
| **Period** | **N** | **N** |  |  | **€** | **€** |  | **€** | **€** |  |
| Pre-index (month -12 to -7) | 4,979 | 9,041 | 7,789 | 14,231 | 11,854 | 7,212 | <0.001 | 6,683 | 5,349 | <0.001 |
| Pre-index (month -6 to -1) | 4,979 | 9,041 | 7,789 | 14,231 | 27,477 | 11,819 | <0.001 | 18,163 | 9,152 | <0.001 |
| Index cost** | 4,979 | 9,041 | 7,789 | 14,231 | 12,884 | 4,543 | <0.001 | 12,856 | 4,509 | <0.001 |
| Month 1-3 | 4,973 | 8,601 | 6,873 | 13,533 | 18,205 | 8,495 | <0.001 | 7,686 | 6,864 | <0.001 |
| Month 4-6 | 3,930 | 7,683 | 5,266 | 12,190 | 11,943 | 5,655 | <0.001 | 4,607 | 4,125 | 0.012 |
| Month 7-9 | 3,478 | 7,291 | 4,888 | 11,610 | 9,938 | 4,587 | <0.001 | 3,541 | 3,349 | 0.670 |
| Month 10-12 | 3,200 | 7,014 | 4,683 | 11,170 | 8,910 | 4,075 | <0.001 | 2,908 | 3,093 | 0.664 |
| Year 2 (month 13-24) | 2,987 | 6,770 | 4,525 | 10,810 | 29,414 | 13,020 | <0.001 | 9,784 | 10,158 | 0.791 |

*P-value from t-test and bootstrapped; **Index admissions are calculated from the cost database for HOHA. Since COHA is partly outpatient onset, their index admission is from DRG

Supplementary Table 7.2. COHA, Total health costs divided into patients with or without complications

|  | **Number of patients** | | | | **Total health cost** | | | | | |
| --- | --- | --- | --- | --- | --- | --- | --- | --- | --- | --- |
|  | **COHA with complications** | | **COHA no complications** | | **COHA with complications** | | | **COHA no complications** | | |
|  | **COHA - complications** | **Control** | **COHA - no complications** | **Control** | **COHA - complications** | **Control** | **P-value** | **COHA - no complications** | **Control** | **P-value** |
| **Period** | **N** | **N** |  |  | **€** | **€** |  | **€** | **€** |  |
| Pre-index (month -12 to -7) | 2,382 | 4,322 | 3,203 | 5,882 | 12,627 | 6,602 | <0.001 | 6,501 | 4,390 | <0.001 |
| Pre-index (month -6 to -1) | 2,382 | 4,322 | 3,203 | 5,882 | 26,903 | 9,270 | <0.001 | 15,736 | 6,279 | <0.001 |
| Index cost** | 2,382 | 4,322 | 3,203 | 5,882 | 4,164 | 3,247 | <0.001 | 3,361 | 2,811 | <0.001 |
| Month 1-3 | 2,382 | 4,151 | 3,189 | 5,717 | 16,986 | 7,336 | <0.001 | 6,954 | 5,491 | <0.001 |
| Month 4-6 | 1,869 | 3,769 | 2,633 | 5,323 | 11,628 | 4,782 | <0.001 | 4,034 | 3,253 | <0.001 |
| Month 7-9 | 1,663 | 3,618 | 2,477 | 5,150 | 9,939 | 4,033 | <0.001 | 3,123 | 2,620 | 0.032 |
| Month 10-12 | 1,535 | 3,502 | 2,403 | 5,013 | 8,569 | 3,739 | <0.001 | 2,852 | 2,361 | 0.021 |
| Year 2 (month 13-24) | 1,437 | 3,388 | 2,345 | 4,917 | 28,779 | 11,958 | <0.001 | 9,201 | 8,398 | 0.215 |

*P-value from t-test and bootstrapped; **Index admissions are calculated from the cost database for HOHA. Since COHA is partly outpatient onset, their index admission is from DRG

Supplementary Table 7.3. HOHA, Total health costs divided into patients with or without complications

|  | **Number of patients** | | | | **Total health cost** | | | | | |
| --- | --- | --- | --- | --- | --- | --- | --- | --- | --- | --- |
|  | **HOHA with complications** | | **HOHA no complications** | | **HOHA with complications** | | | **HOHA no complications** | | |
|  | **HOHA - complications** | **Control** | **HOHA - no complications** | **Control** | **HOHA - complications** | **Control** | **P-value** | **HOHA - no complications** | **Control** | **P-value** |
| **Period** | **N** | **N** |  |  | **€** | **€** |  | **€** | **€** |  |
| Pre-index (month -12 to -7) | 2,597 | 4,719 | 4,586 | 8,349 | 11,145 | 7,770 | <0.001 | 6,810 | 6,025 | 0.006 |
| Pre-index (month -6 to -1) | 2,597 | 4,719 | 4,586 | 8,349 | 28,004 | 14,153 | <0.001 | 19,857 | 11,176 | <0.001 |
| Index cost** | 2,597 | 4,719 | 4,586 | 8,349 | 20,882 | 5,730 | <0.001 | 19,488 | 5,705 | <0.001 |
| Month 1-3 | 2,591 | 4,450 | 3,684 | 7,816 | 19,325 | 9,576 | <0.001 | 8,320 | 7,868 | 0.504 |
| Month 4-6 | 2,061 | 3,914 | 2,633 | 6,867 | 12,228 | 6,496 | <0.001 | 5,179 | 4,801 | 0.544 |
| Month 7-9 | 1,815 | 3,673 | 2,411 | 6,460 | 9,938 | 5,133 | <0.001 | 3,969 | 3,930 | 1.000 |
| Month 10-12 | 1,665 | 3,512 | 2,280 | 6,157 | 9,225 | 4,410 | <0.001 | 2,966 | 3,689 | 0.002 |
| Year 2 (month 13-24) | 1,550 | 3,382 | 2,180 | 5,893 | 30,003 | 14,083 | <0.001 | 10,411 | 11,628 | 0.063 |

*P-value from t-test and bootstrapped; **Index admissions are calculated from the cost database for HOHA. Since COHA is partly outpatient onset, their index admission is from DRG

Survival analysis

The survival analysis followed a case-control design, and the analysis period was 2011-2014. The time unit used in the analysis was months since the initial mortality was rather high, and thus yearly survival would be too crude an estimate.First, we used a Kaplan Meyer analysis to depict the monthly survival curves for case vs controls for:

1. All CDI vs control (Supplementary Table 9)
2. HOHA vs control
3. COHA vs control

We then split the population into four age groups: 0-30 years, 31-50 years, 51-70 years, and 70+ years. A Kaplan Meyer analysis was performed for each age group (Supplementary Figure 1-2).

Supplementary Table 8. Censored patients

|  | **All Patients** | | **HOHA** | | **COHA** | |
| --- | --- | --- | --- | --- | --- | --- |
|  | **CDI** | **Control** | **CDI** | **Control** | **CDI** | **Control** |
| **Number of patients, n** | 12,768 | 23,272 | 7,183 | 13,068 | 5,585 | 10,204 |
|  |  |  |  |  |  |  |
| **Share censored all patients, %** | 39.8 | 56.7 | 32.0 | 49.2 | 50.0 | 66.2 |
| **Age groups, %** |  |  |  |  |  |  |
| 0-30 | 94.4 | 98.2 | 87.0 | 96.9 | 97.7 | 98.7 |
| 31-50 | 80.4 | 90.8 | 72.1 | 83.9 | 86.9 | 95.8 |
| 51-70 | 48.7 | 68.9 | 42.4 | 62.6 | 56.5 | 76.5 |
| 71+ | 25.3 | 43.0 | 21.7 | 38.9 | 31.0 | 49.6 |


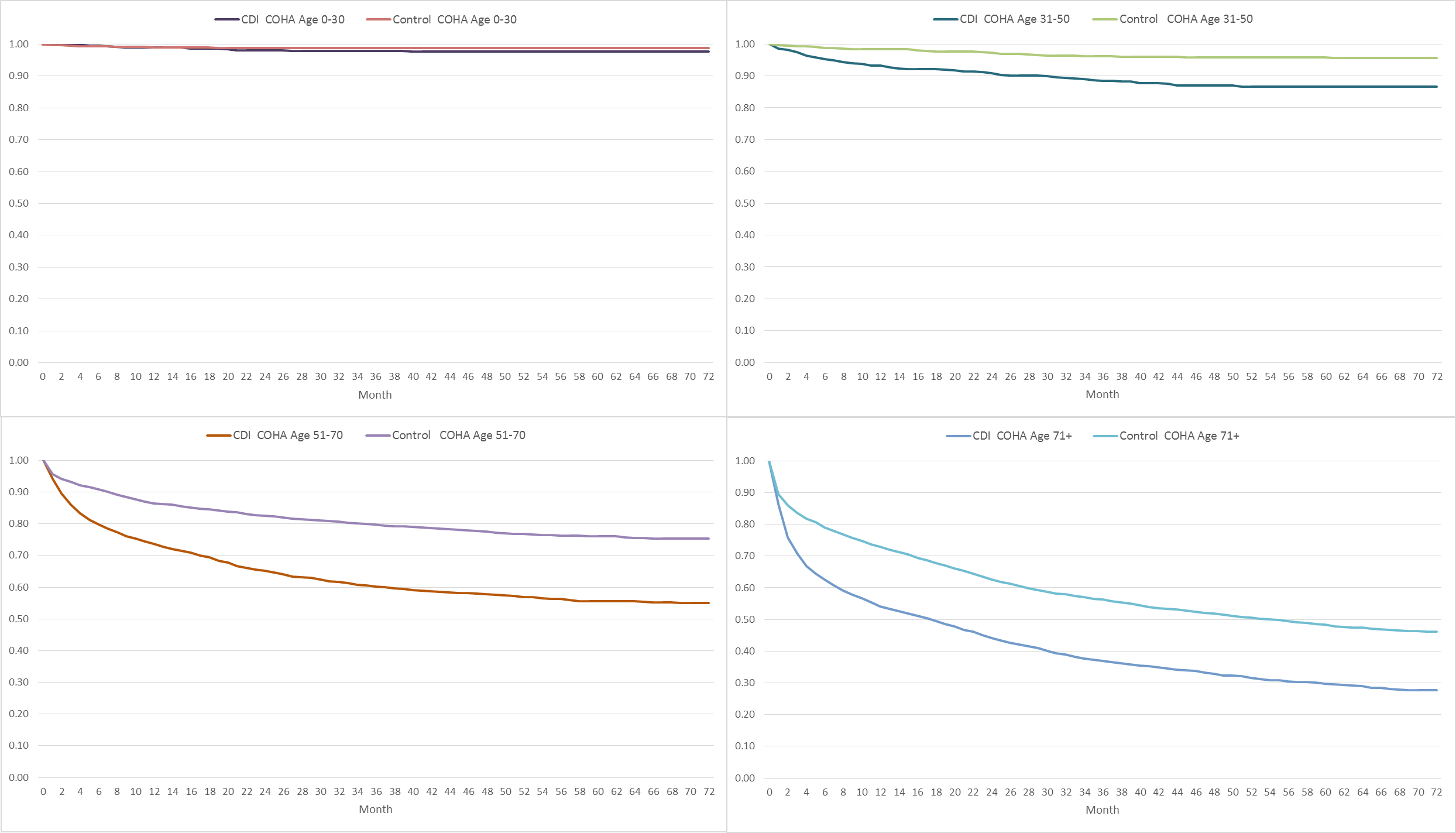


Supplementary Figure 1. Survival curve for COHA patients by age group


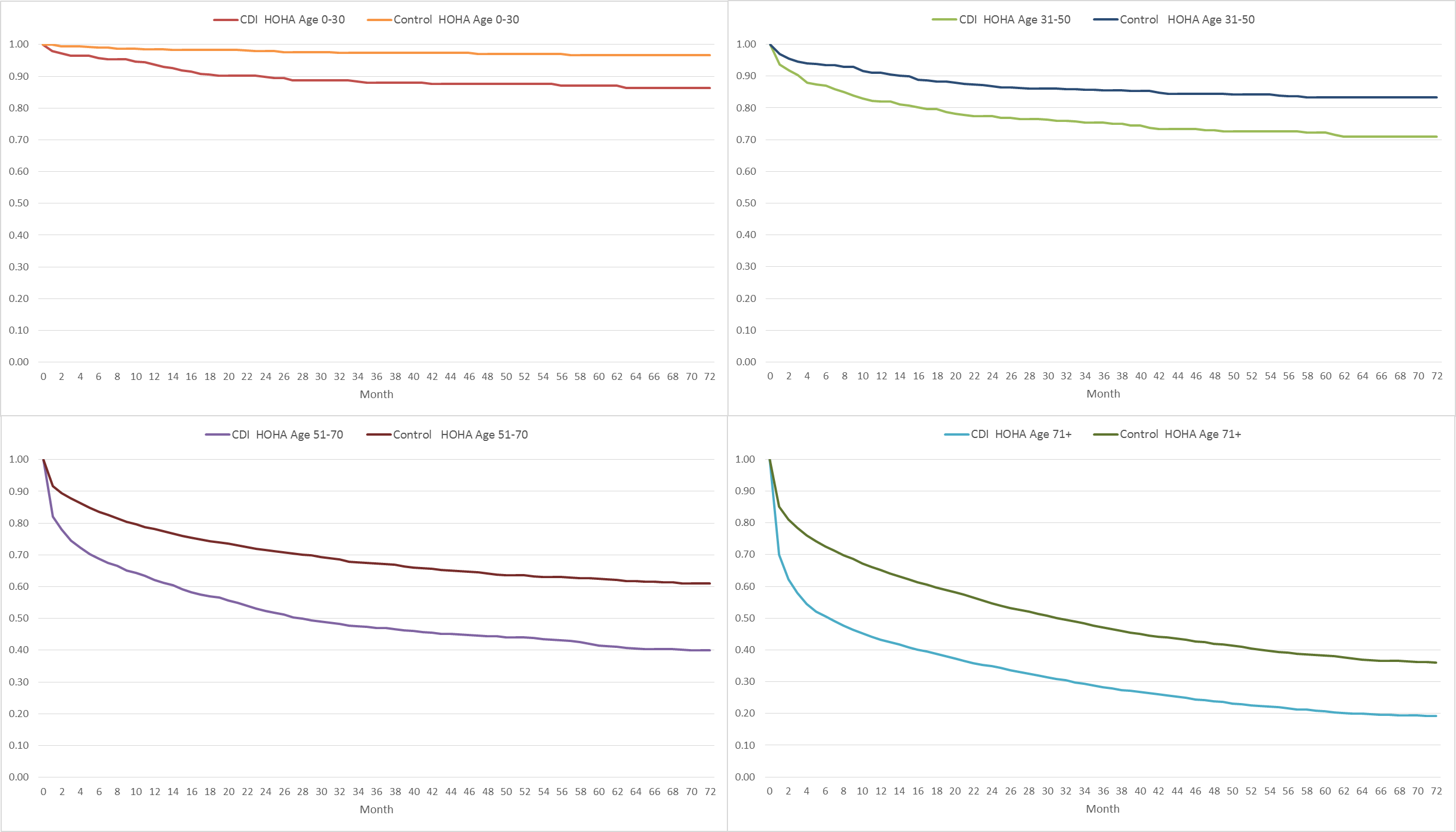


Supplementary Figure2. Survival curve for HOHA patients by age group
